# Incidence of diarrhoeal disease among children aged less than five years in low- and middle-income countries: a systematic review

**DOI:** 10.7189/jogh.15.04107

**Published:** 2025-04-25

**Authors:** Gedefaw Abeje Fekadu, Damen Hailemariam, Firmaye Bogale Woldie, Robera Olana Fite, Kassahun Alemu, Alemayehu Worku, Lisanu Taddesse, Delayehu Bekele, Getachew Tolera, Grace J Chan, Muluemebet Abera

**Affiliations:** 1 Health System and Reproductive Health Research Directorate, Ethiopian Public Health Institute, Addis Ababa, Ethiopia; 2 Department of Reproductive Health and Population Studies, School of Public Health, College of Medicine and Health Sciences, Bahir Dar University, Bahir Dar, Ethiopia; 3 School of Public Health, College of Health Sciences, Addis Ababa University, Addis Ababa, Ethiopia; 4 Knowledge Translation Directorate, Ethiopian Public Health Institute, Addis Ababa, Ethiopia; 5 HaSET Maternal and Child Health Research Program, Addis Ababa, Ethiopia; 6 Department of Obstetrics and Gynaecology, Saint Paul’s Hospital Millennium Medical College, Addis Ababa, Ethiopia; 7 Research and Technology Transfer, Ethiopian Public Health Institute, Addis Ababa, Ethiopia; 8 Department of Epidemiology, Harvard University T. H. Chan School of Public Health, Boston, Massachusetts, USA; 9 Department of Paediatrics, Boston Children's Hospital, Harvard Medical School, Boston, Massachusetts, USA; 10 Department of Population and Family Health, Faculty of Public Health, Institute of Health Science, Jimma University, Jimma, Ethiopia

## Abstract

**Background:**

Diarrhoea was the second leading cause of death among children aged <5 years in 2019. Most of these deaths occurred in low- and middle-income countries (LMICs). Summarising the available up-to-date evidence on the incidence of diarrhoeal disease among children could help track the effectiveness of diarrhoea prevention and control efforts. We summarised available evidence on the incidence of diarrhoea among children aged <5 years in LMICs.

**Methods:**

We included cross-sectional or cohort studies that reported diarrhoeal incidence among children aged <5 years in LMICs that were published between 2010–22 in English. Two authors searched, reviewed the quality of the selected articles, and extracted the data. We searched Medline/Pubmed, Web of Science, Scopus, CINHAL, EMBASE, WorldCat, OpenGrey, dissertations/theses, reports, and Google Scholar. We screened articles by title, abstract, and full text.

**Results:**

We included 15 articles that met the inclusion criteria in the analysis. Four studies were from Africa, seven were from Asia, two were from Brazil, and two were from Nicaragua. Seven studies were conducted in urban settings, six in rural settings, and two in urban and rural areas. The highest incidence of diarrhoea was 5200 episodes of diarrhoea per 1000 child-years, and the lowest was 60.4 episodes of diarrhoea per 1000 child-years.

**Conclusions:**

There is limited evidence on the incidence of diarrhoea among children aged <5 years in LMICs. The available studies identified major differences in the incidence of diarrhoea by country, 60.4 in China, and 5200 episodes of diarrhoea per 1000 infants. We recommend more up-to-date primary studies on the incidence of diarrhoea among children aged <5 years in LMICs to monitor and evaluate the effectiveness of diarrhoea control and prevention policies and interventions.

**Registration:**

PROSPERO: CRD42022290180.

Although under-five mortality has dropped by 60% since 1990, approximately 5.2 million children aged <5 years died in 2019, mostly in low- and middle-income countries (LMICs) [1]. In the same year, diarrhoea was the second leading cause of death for these children [2,3].

The World Health Organization (WHO) defines diarrhoea as the passage of three or more loose or liquid stools per day [2]. Diarrhoea can be acute watery, acute bloody or chronic. Acute diarrhoea lasts several hours or days. Chronic diarrhoea lasts >14 days. Diarrhoea can be classified as severe, moderate and mild based on its severity [2]. It is commonly a symptom of an infection in the intestinal tract, which could be bacterial, viral, or parasitic [2,4,5]. Diarrhoea may cause severe dehydration and lead to malnutrition that increases the risk of death [6,7]. A systematic review in 2012 on the incidence of diarrhoea among children in LMICs estimated 3.4 episodes of diarrhoea per child-year in 1990 and 2.9 episodes per child-year in 2010. That review noted that diarrhoea incidence rates were highest among infants aged six to 11 months (4.5 episodes per child per year) and lowest among children aged 24–59 months (2.3 episodes per child-year) [8].

Updating the information provided in the 2012 published review is needed due to changing circumstances in LMICs that could affect policy decisions or future interventions. Synthesising this up-to-date evidence on the incidence of diarrhoeal disease among children aged <5 years in LMICs could support the planning and implementation of diarrhoea prevention and control efforts. The incidence could be used to estimate the amount of oral rehydration salt or other rehydration therapies/treatments needed to treat children aged <5 years with diarrhoea. This information could also be used in evaluating the effectiveness of diarrhoea prevention and control efforts. Therefore, we conducted a systematic review and summarised available evidence between 2010–22 on the incidence of diarrhoeal disease among children aged <5 years in LMICs.

## METHODS

### Design and setting

We designed, conducted, and reported the protocol for this review using the PRISMA guidelines [9]. We registered the protocol at PROSPERO (registration number CRD42022290180) [10]. We reviewed the manuscript using the 2020 PRISMA checklist (Table S1 in the **Online Supplementary Document**).

### Search strategy, search sources, inclusion, and exclusion criteria

We included cross-sectional or cohort studies that reported the incidence of diarrhoeal disease among children aged <5 years in LMICs published from August 2010 to February 2022 in English. Diarrhoea was defined as the passage of three or more loose, watery stools within 24 hours [2]. LMICs were defined by the World Bank per the country’s gross national income in 2022. Countries with less than USD 1035 gross national income per capita were classified as low-income, those with between USD 1036–4085 as lower middle income, and those between USD 4086–12 615 as upper middle-income countries [11]. We included both published studies and unpublished grey literature. We excluded commentaries, letters, protocols, and editorials.

Two independent reviewers (GA and FB) searched multiple databases, including Scopus, Medline/Pubmed, Web of Science, and EMBASE, to access published articles. We searched unpublished articles (grey literature) from Google Scholar, WorldCat, OpenGrey, and online thesis/dissertation repositories. We used MeSH keywords and free text search terms to identify the articles. We used the following search terms: diarrh(o)ae, incidence, rate, frequency, child/children, childhood, under-five, morbidity, surveillance, burden of disease, developing countries, low-income, middle-income, and the name of each country. We combined these terms with the Boolean operator ‘OR’ to broaden or ‘AND’ to narrow the search (Table S2 in the **Online Supplementary Document**). We screened references of identified articles to identify potential articles not found by searching databases. In addition, we contacted experts, researchers, and relevant organisations for suggestions of relevant articles.

### Study selection

We exported all articles found using our search strategy to EndNote, version 20 (Clarivate Analytics, London, UK). We removed the duplicates, and two authors (GA and FB) screened and reviewed the remaining articles based on the inclusion and exclusion criteria, including the titles, abstracts, and full articles. Disagreements were solved through discussion or by a third reviewer (RO).

### Data extraction and processing

Two independent reviewers (GA and FB) conducted data extraction using a standard data extraction Excel sheet that was pre-tested to check for consistency between reviewers. The data extraction format captured the authors’ names, publication year, study period, sample size, sampling, study setting, country of the study, study design, and diarrhoea incidence among children aged <5 years. Any disagreement in data abstraction between the two authors was resolved by a third independent reviewer (RO). Extracted data was then transferred to Stata, version 17 (StataCorp, College Station, Texas, USA) for analysis.

### Quality appraisal

We used the Newcastle-Ottawa scale quality appraisal checklist to assess the quality of included studies based on three domains – selection of the study groups, comparability of the groups and ascertainment of the exposure/outcome [12]. With a maximum score of nine and 10, studies representing a score <5 were considered at high risk of bias and were not included in the systematic review [2,13].

### Synthesis

We developed a narrative synthesis to characterise the study. Tables and figures displayed characteristics of the included studies. Incidence rates reported in other forms were converted to diarrhoea per 1000 child-years to make the interpretation of incidences less than one more understandable.

### Subgroup synthesis

We completed a subgroup narrative synthesis based on the age of the child. diarrhoea incidence was displayed for children aged zero to 59 months, zero to 11 months, 12–23 months, 24–59 months, six to 48 months, zero to 36 months, 24–36 months, and 18–24 months.

## RESULTS

### Literature search

**Figure 1:**
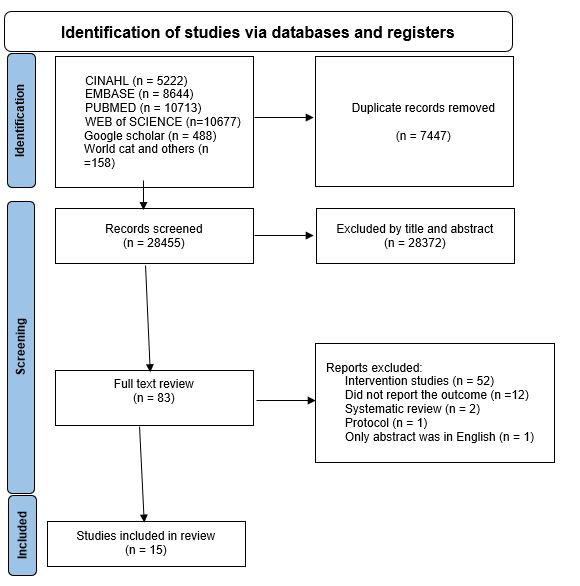
PRISMA flow diagram for selection of articles for systematic review of incidence of diarrhoea among children aged <5 years in low- and middle-income countries, 2010–22.

We identified a total of 35 902 articles, and 7447 articles were duplicates and were removed. Another 28 372 were excluded by title and abstract review. We selected 83 articles for full review. From these, we excluded 52 because they were intervention studies, 12 did not report the outcome, two were systematic reviews, one was a protocol, and one had a non-English language abstract. Finally, we included 15 articles for full review (**Figure 1**).

### Characteristics of selected studies

12 of the included studies [4–7,14–21] were cohort studies by design, whereas the other three [22–24] were cross-sectional. Three were published in 2021 [14,22,24], one was published in 2020 [15], one was published in 2016 [16], three were published in 2015 [17–19], five were published in 2014 [4–7,23], one in 2013 [20], and one in 2011 [21].

Four studies [6,14,22,23] were from Africa, seven studies [5,15,16,18,19,21,24] were from Asia, two studies were from South America [4,17], and two were from Central America [7,20].

By country, four of the studies were from India [5,16,18,21], two from Nicaragua [7,20], two from Brazil [4,17], and the others were from South Africa [22], Pakistan [15], Vietnam [19], Tanzania [23], China [24], Egypt [6], and Malawi [14]. In terms of study setting, seven studies [4,5,7,16,17,20,22] were conducted in an urban setting, six [6,14,15,21,23,24] were in a rural setting, and two reported both urban and rural incidence [18,19].

All the studies used the WHO definition for diarrhoea. All studies reported all forms of diarrhoea except one study [24], which reported only moderate and severe diarrhoea. The duration of follow-up was one year for eight studies [4,5,7,14,17,19–21,24], one and half years for one study [18], two years for one study [6], three years for two studies [15,16], one week for one study [23], and two weeks for one study [22]. Nine studies included children from birth to 59 months [6,7,15,18,20–24], one study included children from six to 48 months [14], one study from birth to three years [16], two studies from birth to 12 months [17,19], and one study from two to 11 months [4].

### Incidence of diarrhoea

Eight studies reported the incidence of diarrhoea among children aged zero to 59 months [7,15,18,20–24]. Two studies reported the incidence of diarrhoea among children aged 24–59 months, which ranged from 13.6 to 480 episodes of diarrhoea per 1000 child-years [18,24]. A study in Malawi reported an incidence of diarrhoea among children aged six to 48 months, which was 200 episodes of diarrhoea per 1000 child-years [14]. Studies in India [16] (1660 episodes of diarrhoea per 1000 child-years) and Egypt [6] (7800 episodes of diarrhoea per 1000 child-years) reported incidences of diarrhoea among children aged from birth to three years. Two studies from India reported the incidence of 940 episodes of diarrhoea per 1000 child-years [16] and 1270 episodes of diarrhoea per 1000 child-years [21] among children aged 24–36 months. Studies in India and Egypt reported an incidence rate of 1090 episodes of diarrhoea per 1000 child-years [5] and 5400 episodes of diarrhoea per 1000 child-years [6] among children aged 18–24 months (**Table 1**).

**Table1:** Incidence of diarrhoea among children aged <5 years in low- and middle-income countries, by country and age of the child, systematic review, 2010–22

Child age, countries, and studies	Incidence of diarrhoea, episodes per 1000 child-years (range)
0–59 months	
*China (Zhending), Zhou et al. 2021 [* 24 *]*	215
*China (Sanjiang), Zhou et al. 2021 [* 24 *]*	398.2
*China (Zhending), Zhou et al. 2021 [* 24 *]*	60.4 (54.2–66.6)
*China (Sanjiang), Zhou et al. 2021 [* 24 *]*	88.3 (78.4–98.2)
*South Africa, Johnstone et al. 2021 [* 22 *]*	1100 (400–2200)
*Pakistan, Hansen et al. 2020 [* 15 *]*	1300 (1000–1500)
*Pakistan, Hansen et al. 2020 [* 15 *]*	700 (600–800)
*Pakistan, Hansen et al. 2020 [* 15 *]*	800 (700–900)
*India, Kattula et al. 2015 [* 18 *]*	510 (440–580)
*India (rural), Kattula et al. 2015 [* 18 *]*	330 (260–420)
*India (urban), Kattula et al. 2015 [* 18 *]*	670 (570–780)
*Tanzania, Mashoto et al. 2014 [* 23 *]*	5200
*Nicaragua, Becker-Dreps et al. 2014 [* 7 *]*	1100
*Nicaragua, Becker-Dreps et al. 2013 [* 20 *]*	358
*Nicaragua, Becker-Dreps et al. 2013 [* 20 *]*	249
*India (Jammu), Lohakpure et al. 2019 [* 21 *]*	1380
0–11 months	
*China (Zhending), Zhou et al. 2021 [* 24 *]*	138.5 (119.0–158.0)
*China (Sanjiang), Zhou et al. 2021 [* 24 *]*	134.0 (106.3–161.7)
*India, Sarkar et al. 2016 [* 16 *]*	2760
*Brazil, da Silva Fernandes Nascimento et al. 2012 [* 17 *]*	320 (210–366)
*India, Kattula et al. 2015 [* 18 *]*	1420 (1080–1860)
*India (rural), Kattula et al. 2015 [* 18 *]*	960 (640–1500)
*India (urban), Kattula et al. 2015 [* 18 *]*	2200 (1500–3100)
*Vietnam, Anders et al. 2015 [* 19 *]*	271
*Vietnam (rural), Anders et al. 2015 [* 19 *]*	604
*Vietnam (urban), Anders et al. 2015 [* 19 *]*	89
*Egypt, Mansour et al. 2014 [* 6 *]*	940
*India (Jammu), Lohakpure et al. 2019 [* 21 *]*	1560
12–23 months	
*China (Zhending), Zhou et al. 2021 [* 24 *]*	102.4 (85.7–119.1)
*China (Sanjiang), Zhou et al. 2021 [* 24 *]*	130.4 (103.9–156.9)
*India, Sarkar et al. 2016 [* 16 *]*	1280
*India, Kattula et al. 2015 [* 18 *]*	510 (420–620)
*India (rural), Kattula et al. 2015 [* 18 *]*	350 (250–480)
*India (urban), Kattula et al. 2015 [* 18 *]*	710 (560–920)
*India (Jammu), Lohakpure et al. 2019 [* 21 *]*	1470
24–59 months	
*China (Zhending), Zhou et al. 2021 [* 24 *]*	13.6 (9.6–17.6)
*China (Sanjiang), Zhou et al. 2021 [* 24 *]*	61.3 (50.7–71.9)
*India, Kattula et al. 2015 [* 18 *]*	330 (260–430
*India (rural), Kattula et al. 2015 [* 18 *]*	70 (30–180)
*India (urban), Kattula et al. 2015 [* 18 *]*	480 (360–610)
6–48 months	
*Malawi, Tizifa et al. 2021 [* 14 *]*	200 (200–300)
0–3 years	
*India, Sarkar et al. 2016 [* 16 *]*	1660
*Egypt, Mansour et al. 2014 [* 6 *]*	7800
24–36 months	
*India, Sarkar et al. 2016 [* 16 *]*	940
*India (Jammu), Lohakpure et al. 2019 [* 21 *]*	1270
2–11 months	
*Brazil (urban), Vieria et al. 2011 [* 4 *]*	500
<6 months	
*India (Bengal, rural), Panda et al. 2014 [* 5 *]*	1020 (801–1303)
6–12 months	
*India (Bengal, rural), Panda et al. 2014 [* 5 *]*	1140 (876–1459)
12–18 months	
*India (Bengal, rural), Panda et al. 2014 [* 5 *]*	860 (650–1108)
*Egypt, Mansour et al. 2014 [* 6 *]*	7100
18–24 months	
*India (Bengal, rural), Panda et al. 2014 [* 5 *]*	1090 (772–1498)
*Egypt, Mansour et al. 2014 [* 6 *]*	5400
37–48 months	
*India (Jammu), Lohakpure et al. 2019 [* 21 *]*	1230
49–60 months	
*India (Jammu), Lohakpure et al. 2019 [* 21 *]*	1380

The highest incidence of diarrhoea reported among children aged <5 years was 5200 episodes of diarrhoea per 1000 child-years from Tanzania [23]. The lowest was 60.4 episodes of diarrhoea per 1000 child-years reported from a study conducted in Zenghding, China [24].

Seven studies reported the incidence of diarrhoea among children aged zero to 11 months [6,16–19,21,24]. The incidence of diarrhoea in this age group ranged from 89.4 per 1000 child-years in a study conducted in urban Vietnam [19] to 2760 episodes per 1000 child-year in a study conducted in India [16]. Four studies reported the incidence of diarrhoea among children aged 12–23 months [16,18,21,24]. According to these studies, the incidence of diarrhoea among children aged 12–23 months ranged from 102.4 [24] to 1470 diarrhoea episodes per 1000 child-years [21].

## DISCUSSION

The incidence of diarrhoea among children aged <5 years in LMICs ranged from 60.4 to 5200 episodes of diarrhoea per 1000 child-years [23,24]. Most of these studies were from Asia, including China and India.

We found incidence rates computed for all children aged <5 years. The majority of studies reported the incidence of diarrhoea among children from zero to 59 months compared to other age-specific incidence rates. Incidence rates were reported for other specific age groups, with seven studies reporting incidence rates for children aged zero to 11 months.

We identified major differences in incidence by country [23,24]. The average incidence of diarrhoea among children aged <5 years old was 903 episodes of diarrhoea per 1000 child years. This was lower than an estimate for LMICs calculated for 2010 that was 2900 episodes of diarrhoea per 1000 child year [8]. However, the 2010 figure was based on model estimation that might have involved a time difference. The incidence of diarrhoea among children aged zero to 11 months ranged from 89.4 episodes of diarrhoea per 1000 child-years in a study conducted in Vietnam [19] to 2700 episodes of diarrhoea per 1000 child-years from a study conducted in India [25].

The mean incidence rate of diarrhoea among children aged zero to 11 months was 777 diarrhoea episodes per 1000 child years. This was lower than the mean incidence of diarrhoea among children aged zero to 59 months. This was in line with studies conducted in LMICs, which showed that the incidence of diarrhoea was lower among children aged zero to 11 months compared to children aged zero to 59 months [8].

The integrated Global Action Plan for the Prevention and Control of Pneumonia and Diarrhoea, was proposed to reduce the incidence of severe diarrhoea among children aged <5 years by 75% compared to the 2010 level by 2025 [26]. The high incidence rate identified in this review poses questions about the achievement of this target.

In this review, we searched many databases to identify all relevant literature, including grey literature. The quality of included articles was evaluated using standard quality assessment checklists. The limitation of this review is that we identified limited evidence on the incidence of diarrhoea among children aged <5 years during the review period. Most of the studies were from Asian countries, with a few from Africa. Some of the incidence reported was based on short person-time observation, which can be overestimated when converted to person-years. We were not able to conduct a meta-analysis due to the heterogeneity of the reported incidence of diarrheal episodes.

## CONCLUSIONS

There is limited evidence on the incidence of diarrhoea among children aged <5 years in LMICs. Most of the available studies are from Asian countries. The available studies have shown a large variation in the incidence of diarrhoea. LMICs need up-to-date data about the incidence of diarrhoea to monitor and evaluate the effectiveness of policies and interventions being implemented to prevent and control diarrhoea [26]. To achieve child health-related targets, we recommend more research to generate timely and accurate estimates of the incidence of diarrhoea among children aged <5 years globally.

## Additional material


Online Supplementary Document
