# Supplementary material for: Incidence of diarrhoeal disease among children aged less than five years in low- and middle-income countries: a systematic review
Source: J Glob Health. 2025 Apr 25;15:04107. doi: 10.7189/jogh.15.04107 (PMC12023804; doi:10.7189/jogh.15.04107)
Supplement: Online Supplementary Document [file jogh-15-04107-s001.pdf]

**Supplement to: Fekadu GA, Hailemariam D, Woldie FB, Fite RO, Alemu K, Worku A, Taddesse L,Bekele D, Tolera G, Chan GJ, Abera M. Incidence of diarrhoeal disease among children aged lessthan five years in low- and middle-income countries: a systematic review. J Glob Health. 2025;15:04107.**

# Table S1:PRISMA 2020 Checklist

| Section and Topic | Item # | Checklist item | Location where item is reported |
|---|---|---|---|
| **TITLE** | | | |
| Title | 1 | Identify the report as a systematic review. | Page 1 |
| **ABSTRACT** | | | |
| Abstract | 2 | See the PRISMA 2020 for Abstracts checklist. – | The abstract is prepared in accordance with the 2020 PRISMA checklist for abstracts (pages 3-4, lines 51-77 |
| **INTRODUCTION** | | | |
| Rationale | 3 | Describe the rationale for the review in the context of existing knowledge. | Page 3 |
| Objectives | 4 | Provide an explicit statement of the objective(s) or question(s) the review addresses. | Page 3 |
| **METHODS** | | | |
| Eligibility criteria | 5 | Specify the inclusion and exclusion criteria for the review and how studies were grouped for the syntheses. | Page 4 |
| Information sources | 6 | Specify all databases, registers, websites, organisations, reference lists and other sources searched or consulted to identify studies. Specify the date when each source was last searched or consulted. | Page 4 |
| Search strategy | 7 | Present the full search strategies for all databases, registers and websites, including any filters and limits used. | Search strategy for PubMed is attached as additional file. |
| Selection process | 8 | Specify the methods used to decide whether a study met the inclusion criteria of the review, including how many reviewers screened each record and each report retrieved, whether they worked independently, and if applicable, details of automation tools used in the process. | Pages 5 |
| Data collection process | 9 | Specify the methods used to collect data from reports, including how many reviewers collected data from each report, whether they worked independently, any processes for obtaining or confirming data from study investigators, and if applicable, details of automation tools used in the process. | Pages 5 |
| Data items | 10a | List and define all outcomes for which data were sought. Specify whether all results that were compatible with each outcome domain in each study were sought (e.g. for all measures, time points, analyses), and if not, the methods used to decide which results to collect. | Pages 5 |
| | 10b | List and define all other variables for which data were sought (e.g. participant and intervention characteristics, funding sources). Describe any assumptions made about any missing or unclear information. | Page 5 |
| Study risk of bias assessment | 11 | Specify the methods used to assess risk of bias in the included studies, including details of the tool(s) used, how many reviewers assessed each study and whether they worked independently, and if applicable, details of automation tools used in the process. | Pages 5 |
| Effect measures | 12 | Specify for each outcome the effect measure(s) (e.g. risk ratio, mean difference) used in the synthesis or presentation of results. | Effect measure was not computed, It was synthesis of the results. |
| Synthesis methods | 13a | Describe the processes used to decide which studies were eligible for each synthesis (e.g. tabulating the study intervention characteristics and comparing against the planned groups for each synthesis (item #5)). | Page 6 |
| | 13b | Describe any methods required to prepare the data for presentation or synthesis, such as handling of missing summary statistics, or data conversions. | Page 6 |
| | 13c | Describe any methods used to tabulate or visually display results of individual studies and syntheses. | Page 6 |

| Section and Topic | Item # | Checklist item | Location where item is reported |
|---|---|---|---|
| | 13d | Describe any methods used to synthesize results and provide a rationale for the choice(s). If meta-analysis was performed, describe the model(s), method(s) to identify the presence and extent of statistical heterogeneity, and software package(s) used. | Page 6 |
| | 13e | Describe any methods used to explore possible causes of heterogeneity among study results (e.g. subgroup analysis, meta-regression). | Page 6 |
| | 13f | Describe any sensitivity analyses conducted to assess robustness of the synthesized results. | Not applicable since we did only systematic review |
| Reporting bias assessment | 14 | Describe any methods used to assess risk of bias due to missing results in a synthesis (arising from reporting biases). | Page 6 |
| Certainty assessment | 15 | Describe any methods used to assess certainty (or confidence) in the body of evidence for an outcome. | Page 6 |
| **RESULTS** | | | |
| Study selection | 16a | Describe the results of the search and selection process, from the number of records identified in the search to the number of studies included in the review, ideally using a flow diagram. | Pages 6 |
| | 16b | Cite studies that might appear to meet the inclusion criteria, but which were excluded, and explain why they were excluded. | Page 6 |
| Study characteristics | 17 | Cite each included study and present its characteristics. | Pages 6 |
| Risk of bias in studies | 18 | Present assessments of risk of bias for each included study. | Pages 6 |
| Results of individual studies | 19 | For all outcomes, present, for each study: (a) summary statistics for each group (where appropriate) and (b) an effect estimates and its precision (e.g. confidence/credible interval), ideally using structured tables or plots. | Pages 6 |
| Results of syntheses | 20a | For each synthesis, briefly summarise the characteristics and risk of bias among contributing studies. | Pages 7 |
| | 20b | Present results of all statistical syntheses conducted. If meta-analysis was done, present for each the summary estimate and its precision (e.g. confidence/credible interval) and measures of statistical heterogeneity. If comparing groups, describe the direction of the effect. | No statistical synthesis |
| | 20c | Present results of all investigations of possible causes of heterogeneity among study results. | not applicable |
| | 20d | Present results of all sensitivity analyses conducted to assess the robustness of the synthesized results. | Not applicable |
| Reporting biases | 21 | Present assessments of risk of bias due to missing results (arising from reporting biases) for each synthesis assessed. | Not applicable |
| Certainty of evidence | 22 | Present assessments of certainty (or confidence) in the body of evidence for each outcome assessed. | Not applicable |
| **DISCUSSION** | | | |
| Discussion | 23a | Provide a general interpretation of the results in the context of other evidence. | Page 8 & 9 |
| | 23b | Discuss any limitations of the evidence included in the review. | Page 9 |
| | 23c | Discuss any limitations of the review processes used. | No limitation |
| | 23d | Discuss implications of the results for practice, policy, and future research. | Page 9 |
| **OTHER INFORMATION** | | | |
| Registration and protocol | 24a | Provide registration information for the review, including register name and registration number, or state that the review was not registered. | Pages 1 & 3 |

| Section and Topic | Item # | Checklist item | Location where item is reported |
|---|---|---|---|
| | 24b | Indicate where the review protocol can be accessed, or state that a protocol was not prepared. | Pages 1 & 3 |
| | 24c | Describe and explain any amendments to information provided at registration or in the protocol. | No amendments was done |
| Support | 25 | Describe sources of financial or non-financial support for the review, and the role of the funders or sponsors in the review. | Page 10 |
| Competing interests | 26 | Declare any competing interests of review authors. | Page 10 |
| Availability of data, code and other materials | 27 | Report which of the following are publicly available and where they can be found: template data collection forms; data extracted from included studies; data used for all analyses; analytic code; any other materials used in the review. | Page 10 |

*From:* Page MJ, McKenzie JE, Bossuyt PM, Boutron I, Hoffmann TC, Mulrow CD, et al. The PRISMA 2020 statement: an updated guideline for reporting systematic reviews. BMJ 2021;372:n71. doi: 10.1136/bmj.n71
For more information, visit: http://www.prisma-statement.org/

Table S2.Logic Grid of search and MeSH terms ofPubMed for systematic review of incidence of diarrhea among under-five children in low- and Middle-income countries.

| Condition | Context | | Population |
|---|---|---|---|
| Text words | Text words | | Text words |
| • Diarrhea | • Low-income countries | • Afghanistan | • Under-five children |
| • Diarrhoea | • Middle income countries | • Guinea-Bissau | • Child |
| • Dysentery | • Developing countries | • Somalia | • Infant |
| • Acute diarrheal disease | • Least Developed Countries | • Burkina Faso | • Preschool |
| • Prevalence | • Less Developed Countries | • Korea | • Toddler |
| • Incidence | • Third-World Countries | • South Sudan | • Childhood |
| • Incidence study | • Low-Income Countries | • Burundi | |
| • Incidence rate | • Central African Republic | • Liberia | |
| • Cumulative incidence | • Yemen | • Sudan | |
| • Frequency | • Ethiopia | • Madagascar | |
| • Surveillance | • Niger | • Syria | |
| • Burdon of disease | • Gambia | • Chad | |
| • Acute diarrhea | • Rwanda | • Malawi | |
| • Acute Diarrheal Disease in Children | • Guinea | • Togo | |
| | • Sierra Leone | • Congo | |
| | • Angola | • Dem. Rep | |
| | • Honduras | • Mali | |
| | • Philippines | • Uganda | |
| | • Algeria | • Eritrea | |
| | • India | • Mozambique | |
| | • Samoa | • Ethiopia | |
| | • Bangladesh | • Niger | |
| | • Indonesia | • Gambia | |
| | • São Tomé | • Rwanda | |
| | • Principe | • Guinea | |
| | • Belize | • Sierra Leone | |
| | • Iran | • Angola | |
| | • Senegal | • Honduras | |
| | • Benin | • Philippines | |
| | • Kenya | • Algeria | |
| | • Solomon Islands | • India | |
| | • Bhutan | • Samoa | |
| | • Kiribati | • Bangladesh | |
| | • Sri Lanka | • Indonesia | |
| | | • São Tomé | |

| | | | |
|---|---|---|---|
| | • Bolivia<br>• Kyrgyz Republic<br>• Lao PDR<br>• Tajikistan<br>• Cambodia<br>• Lesotho<br>• Timor-Leste<br>• Cameroon<br>• Mauritania<br>• Tunisia<br>• Comoros<br>• Micronesia<br>• Ukraine<br>• Congo<br>• Mongolia<br>• Uzbekistan<br>• Côte d'Ivoire<br>• Morocco<br>• Vanuatu<br>• Djibouti<br>• Myanmar<br>• Vietnam<br>• Egypt<br>• Nepal<br>• West Bank<br>• Gaza<br>• Brazil<br>• Kazakhstan<br>• South Africa<br>• Bulgaria<br>• Kosovo<br>• St. Lucia<br>• China<br>• Lebanon<br>• St. Vincent<br>• The Grenadines<br>• Colombia<br>• Libya<br>• Suriname<br>• Costa Rica<br>• Malaysia<br>• Dominican Republic<br>• Mauritius<br>• Turkmenistan | • Principe<br>• Tanzania<br>• Cabo Verde<br>• Eswatini<br>• Nigeria<br>• Zimbabwe<br>• Ghana<br>• Pakistan<br>• Haiti<br>• Papua New Guinea<br>• Albania<br>• Gabon<br>• Namibia<br>• American Samoa<br>• Georgia<br>• North Macedonia<br>• Argentina<br>• Grenada<br>• Panama<br>• Armenia<br>• Guatemala<br>• Paraguay<br>• El Salvador<br>• Nicaragua<br>• Zambia<br>• Azerbaijan<br>• Guyana<br>• Peru<br>• Belarus<br>• Iraq<br>• Romania<br>• Bosnia<br>• Herzegovina<br>• Jamaica<br>• Russia<br>• Botswana<br>• Jordan<br>• Serbia<br>• Thailand<br>• Cuba<br>• Maldives<br>• Tonga<br>• Dominica | |

| | | | |
|---|---|---|---|
| | • Equatorial Guinea<br>• Mexico<br>• Tuvalu<br>• Ecuador<br>• Moldova<br>• Fiji<br>• Montenegro | • Marshall Islands<br>• Turkey<br>• Equatorial Guinea<br>• Mexico<br>• Tuvalu<br>• Ecuador | |
| **MeSH terms**<br><br>• Diarrhea<br>• Diarrhea/epidemiology<br>• Epidemiology<br>• Public Health Surveillance<br>• Population Surveillance<br>• Epidemiological Monitoring<br>• Diarrhea, Infantile<br>• Gastroenteritis<br>• Dysentery<br>• Gastrointestinal Diseases | **MeSH terms**<br><br>• Low-income countries<br>• Middle income countries<br>• Developing countries<br>• Least Developed Countries<br>• Less Developed Countries<br>• Third-World Countries<br>• Low-Income Countries<br>• Afghanistan<br>• Guinea-Bissau<br>• Somalia<br>• Burkina Faso<br>• Korea<br>• South Sudan<br>• Burundi<br>• Liberia<br>• Sudan<br>• Central African Republic<br>• Madagascar<br>• Syria<br>• Chad<br>• Malawi<br>• Togo<br>• Congo<br>• Dem. Rep<br>• Mali<br>• Uganda<br>• Eritrea<br>• Mozambique<br>• Yemen<br>• Ethiopia<br>• Niger<br>• Gambia<br>• Rwanda<br>• Guinea | •<br>• Tunisia<br>• Comoros<br>• Micronesia<br>• Ukraine<br>• Congo<br>• Mongolia<br>• Uzbekistan<br>• Côte d'Ivoire<br>• Morocco<br>• Vanuatu<br>• Djibouti<br>• Myanmar<br>• Vietnam<br>• Egypt<br>• Nepal<br>• West Bank<br>• Gaza<br>• El Salvador<br>• Nicaragua<br>• Zambia<br>• Eswatini<br>• Nigeria<br>• Zimbabwe<br>• Ghana<br>• Pakistan<br>• Haiti<br>• Papua New Guinea<br>• Albania<br>• Gabon<br>• Namibia<br>• American Samoa<br>• Georgia<br>• North Macedonia<br>• Argentina<br>• Grenada | **MeSH terms**<br><br>• Child<br>• Infant<br>• Child, preschool |

| | | | |
|---|---|---|---|
| | • Sierra Leone<br>• Angola<br>• Honduras<br>• Philippines<br>• Algeria<br>• India<br>• Samoa<br>• Bangladesh<br>• Indonesia<br>• São Tomé<br>• Principe<br>• Belize<br>• Iran<br>• Senegal<br>• Benin<br>• Kenya<br>• Solomon Islands<br>• Bhutan<br>• Kiribati<br>• Sri Lanka<br>• Bolivia<br>• Kyrgyz Republic<br>• Tanzania<br>• Cabo Verde<br>• Lao PDR<br>• Tajikistan<br>• Cambodia<br>• Lesotho<br>• Timor-Leste<br>• Cameroon<br>• Mauritania<br>• Equatorial Guinea<br>• Mexico<br>• Tuvalu<br>• Ecuador<br>• Moldova<br>• Fiji<br>• Montenegro<br>• Dominican Republic<br>• Mauritius<br>• Turkmenistan | • Panama<br>• Armenia<br>• Guatemala<br>• Paraguay<br>• Azerbaijan<br>• Guyana<br>• Peru<br>• Belarus<br>• Iraq<br>• Romania<br>• Bosnia<br>• Herzegovina<br>• Jamaica<br>• Russia<br>• Botswana<br>• Jordan<br>• Serbia<br>• Brazil<br>• Kazakhstan<br>• South Africa<br>• Bulgaria<br>• Kosovo<br>• St. Lucia<br>• China<br>• Lebanon<br>• St. Vincent<br>• The Grenadines<br>• Colombia<br>• Libya<br>• Suriname<br>• Costa Rica<br>• Malaysia<br>• Thailand<br>• Cuba<br>• Maldives<br>• Tonga<br>• Dominica<br>• Marshall Islands<br>• Turkey | |

**Search history results from of PubMed** for systematic review of incidence of diarrhea among under-five children in low- and Middle-income countries.

| Search # | Search terms (filter applied *From 2010/9/1 to 2022/2/21)* | Search results | Date & time |
|---|---|---|---|
| #1 | (((((((((((((((((((((((Diarrhea) OR (Diarrhoea)) OR (Dysentery)) OR (Acute diarrheal disease)) OR (Prevalence)) OR (Incidence)) OR (Incidence study)) OR (Incidence rate)) OR (Cumulative incidence)) OR (Frequency)) OR (Surveillance)) OR (Burdon of disease)) OR (Acute diarrhea)) OR (Acute Diarrheal Disease in Children) ) OR (Diarrhea[MeSH Terms])) OR (Diarrhea/epidemiology[MeSH Terms])) OR (Epidemiology[MeSH Terms])) OR (Public Health Surveillance[MeSH Terms])) OR (Population Surveillance[MeSH Terms])) OR (Epidemiological Monitoring[MeSH Terms])) OR (Diarrhea, Infantile[MeSH Terms])) OR (Gastroenteritis[MeSH Terms])) OR (Dysentery[MeSH Terms])) OR (Gastrointestinal Diseases[MeSH Terms]) | 3,405,425 | February 22, 2022<br><br>02:22:49 |
| #2 | ((((((((((((((((((((((((((((((((((((((((((((((((((((((((((((((((((((((((((((((((((((((((((((((((((((((((((((((((((((((((((((((((((((((((((((((((((((((((((((((((((((((((((((((((((((((((((((((((((((((((((((((((((((((((((((((((((((((((((((((((((((((((((((((((((((((((((((((((((((((((((((((((((((((((((((((((((((Low-income countries) OR (Middle income countries)) OR (Developing countries)) OR (Least Developed Countries)) OR (Less Developed Countries)) OR (Third-World Countries)) OR (Low-Income Countries)) OR (Central African Republic)) OR (Yemen)) OR (Ethiopia)) OR (Niger)) OR (Gambia)) OR (Rwanda)) OR (Guinea)) OR (Sierra Leone)) OR (Angola)) OR (Honduras)) OR (Philippines)) OR (Algeria)) OR (India)) OR (Samoa)) OR (Bangladesh)) OR (Indonesia)) OR (São Tomé)) OR (Principe)) OR (Belize)) OR (Iran)) OR (Senegal)) OR (Benin)) OR (Kenya)) OR (Solomon Islands)) OR (Bhutan)) OR (Kiribati)) OR (Sri Lanka)) OR (Bolivia)) OR (Kyrgyz Republic)) OR (Lao PDR)) OR (Tajikistan)) OR (Cambodia)) OR (Lesotho)) OR (Timor-Leste)) OR (\)) OR (Cameroon)) OR (Mauritania)) OR (Tunisia)) OR (Comoros)) OR (Micronesia)) OR (Ukraine)) OR (Congo)) OR (Mongolia)) OR (Uzbekistan)) OR (Côte d'Ivoire)) OR (Morocco)) OR (Vanuatu)) OR (Djibouti)) OR (Myanmar)) OR (Vietnam)) OR (Egypt)) OR (Nepal)) OR (West Bank)) OR (Gaza)) OR (Brazil)) OR (Kazakhstan)) OR (South Africa)) OR (Bulgaria)) OR (Kosovo)) OR (St. Lucia)) OR (China)) OR (Lebanon)) OR (St. Vincent)) OR (The | 4,514,596 | February 22, 2022<br><br>02:25:45 |

| | | | |
|---|---|---|---|
| | **Grenadines)) OR (Colombia)) OR (Libya)) OR (Suriname)) OR (Costa Rica)) OR (Malaysia)) OR (Dominican Republic)) OR (Mauritius)) OR (Turkmenistan)) OR (Equatorial Guinea)) OR (Mexico)) OR (Tuvalu)) OR (Ecuador)) OR (Moldova)) OR (Fiji)) OR (Montenegro)) OR (Afghanistan)) OR (Guinea-Bissau)) OR (Somalia)) OR (Burkina Faso)) OR (Korea)) OR (South Sudan)) OR (Burundi)) OR (Liberia)) OR (Sudan)) OR (Madagascar)) OR (Syria)) OR (Chad)) OR (Malawi)) OR (Togo)) OR (Congo)) OR (Dem. Rep)) OR (Mali)) OR (Uganda)) OR (Eritrea)) OR (Mozambique)) OR (Ethiopia)) OR (Niger)) OR (Gambia)) OR (Rwanda)) OR (Guinea Sierra Leone)) OR (Angola)) OR (Honduras)) OR (Philippines)) OR (Algeria)) OR (India)) OR (Samoa)) OR (Bangladesh)) OR (Indonesia)) OR (São Tomé)) OR (Principe)) OR (Tanzania)) OR (Cabo Verde)) OR (Eswatini)) OR (Nigeria)) OR (Zimbabwe)) OR (Ghana)) OR (Pakistan)) OR (Haiti)) OR (Papua New Guinea)) OR (Albania)) OR (Namibia)) OR (American Samoa)) OR (Georgia)) OR (Gabon)) OR (North Macedonia)) OR (Argentina)) OR (Grenada)) OR (Panama)) OR (Armenia)) OR (Guatemala)) OR (Paraguay)) OR (El Salvador)) OR (Nicaragua)) OR (Zambia)) OR (Azerbaijan)) OR (Guyana)) OR (Peru)) OR (Belarus)) OR (Iraq)) OR (Romania)) OR (Bosnia)) OR (Herzegovina)) OR (Jamaica)) OR (Russia)) OR (Botswana)) OR (Jordan)) OR (Serbia)) OR (Thailand)) OR (Cuba)) OR (Maldives)) OR (Tonga)) OR (Dominica)) OR (Marshall Islands)) OR (Turkey)) OR (Equatorial Guinea)) OR (Mexico)) OR (Tuvalu Ecuador)) OR (Low-income countries[MeSH Terms])) OR (Middle income countries[MeSH Terms])) OR (Developing countries[MeSH Terms])) OR (Least Developed Countries[MeSH Terms])) OR (Less Developed Countries[MeSH Terms])) OR (Third-World Countries[MeSH Terms])) OR (Low-Income Countries[MeSH Terms])) OR (Afghanistan[MeSH Terms])) OR (Albania[MeSH Terms])) OR (Algeria[MeSH Terms])) OR (Argentina[MeSH Terms])) OR (Armenia[MeSH Terms])) OR (Angola[MeSH Terms])) OR (American Samoa[MeSH Terms])) OR (Azerbaijan[MeSH Terms])) OR (Bangladesh[MeSH Terms])) OR (Belarus[MeSH Terms])) OR (Belize[MeSH Terms])) OR (Benin[MeSH Terms])) OR (Bhutan[MeSH Terms])) OR (Bolivia[MeSH Terms])) OR (Bosnia[MeSH Terms])) OR (Botswana[MeSH Terms])) OR (Brazil[MeSH Terms])) OR (Bulgaria[MeSH Terms])) OR (Burkina Faso[MeSH Terms])) OR (Burundi[MeSH Terms])) OR (Cabo Verde[MeSH Terms])) OR (Cambodia[MeSH Terms])) OR (Cameroon[MeSH Terms])) OR (Central African Republic[MeSH Terms])) OR (Chad[MeSH Terms])) OR (China[MeSH Terms])) OR (Colombia[MeSH Terms])) OR (Comoros[MeSH Terms])) OR (Congo[MeSH Terms])) OR (Congo[MeSH Terms])) OR (Costa Rica[MeSH Terms])) OR (Côte d'Ivoire[MeSH Terms])) OR (Cuba[MeSH Terms])) OR (Dem. Rep[MeSH Terms])) OR (Djibouti[MeSH Terms])) OR (Dominica[MeSH Terms])) OR** | | |

| | | | |
|---|---|---|---|
| | **(Dominican Republic[MeSH Terms])) OR (Ecuador[MeSH Terms])) OR (Egypt[MeSH Terms])) OR (El Salvador[MeSH Terms])) OR (Equatorial Guinea[MeSH Terms])) OR (Eritrea[MeSH Terms])) OR (Eswatini[MeSH Terms])) OR (Ethiopia[MeSH Terms])) OR (Fiji[MeSH Terms])) OR (Gabon[MeSH Terms])) OR (Gambia[MeSH Terms])) OR (Gaza[MeSH Terms])) OR (Georgia[MeSH Terms])) OR (Ghana[MeSH Terms])) OR (Grenada[MeSH Terms])) OR (Guatemala[MeSH Terms])) OR (Guinea[MeSH Terms])) OR (Guinea-Bissau[MeSH Terms])) OR (Guyana[MeSH Terms])) OR (Haiti[MeSH Terms])) OR (Herzegovina[MeSH Terms])) OR (Honduras[MeSH Terms])) OR (India[MeSH Terms])) OR (Indonesia[MeSH Terms])) OR (Iran[MeSH Terms])) OR (Iraq[MeSH Terms])) OR (Jamaica[MeSH Terms])) OR (Jordan[MeSH Terms])) OR (Kazakhstan[MeSH Terms])) OR (Kenya[MeSH Terms])) OR (Kiribati[MeSH Terms])) OR (Korea[MeSH Terms])) OR (Kosovo[MeSH Terms])) OR (Kyrgyz Republic[MeSH Terms])) OR (Lao PDR[MeSH Terms])) OR (Lebanon[MeSH Terms])) OR (Lesotho[MeSH Terms])) OR (Liberia[MeSH Terms])) OR (Libya[MeSH Terms])) OR (Madagascar[MeSH Terms])) OR (Malawi[MeSH Terms])) OR (Malaysia[MeSH Terms])) OR (Maldives[MeSH Terms])) OR (Mali[MeSH Terms])) OR (Marshall Islands[MeSH Terms])) OR (Mauritania[MeSH Terms])) OR (Mauritius[MeSH Terms])) OR (Mexico[MeSH Terms])) OR (Micronesia[MeSH Terms])) OR (Moldova[MeSH Terms])) OR (Mongolia[MeSH Terms])) OR (Montenegro[MeSH Terms])) OR (morocco[MeSH Terms])) OR (Mozambique[MeSH Terms])) OR (Myanmar[MeSH Terms])) OR (Namibia[MeSH Terms])) OR (Nepal[MeSH Terms])) OR (Nicaragua[MeSH Terms])) OR (Niger[MeSH Terms])) OR (Nigeria[MeSH Terms])) OR (North Macedonia[MeSH Terms])) OR (Pakistan[MeSH Terms])) OR (Panama[MeSH Terms])) OR (Papua New Guinea[MeSH Terms])) OR (Paraguay[MeSH Terms])) OR (Peru[MeSH Terms])) OR (Philippines[MeSH Terms])) OR (Principe[MeSH Terms])) OR (Romania[MeSH Terms])) OR (Russia[MeSH Terms])) OR (Rwanda[MeSH Terms])) OR (Samoa[MeSH Terms])) OR (São Tomé[MeSH Terms])) OR (Senegal[MeSH Terms])) OR (Serbia[MeSH Terms])) OR (Sierra Leone[MeSH Terms])) OR (Solomon Islands[MeSH Terms])) OR (Somalia[MeSH Terms])) OR (South Africa[MeSH Terms])) OR (South Sudan[MeSH Terms])) OR (St. Lucia[MeSH Terms])) OR (St. Vincent[MeSH Terms])) OR (Sudan[MeSH Terms])) OR (Suriname[MeSH Terms])) OR (Sri Lanka[MeSH Terms])) OR (Syria[MeSH Terms])) OR (Tajikistan[MeSH Terms])) OR (Tanzania[MeSH Terms])) OR (Thailand[MeSH Terms])) OR (The Grenadines[MeSH Terms])) OR (Timor-Leste[MeSH Terms])) OR (Togo[MeSH Terms])) OR (Tonga[MeSH Terms])) OR (Tunisia[MeSH Terms])) OR (Turkey[MeSH Terms])) OR (Turkmenistan[MeSH Terms])) OR** | | |

| | | | |
|---|---|---|---|
| | **(Tuvalu[MeSH Terms])) OR (Uganda[MeSH Terms])) OR (Ukraine[MeSH Terms])) OR (Uzbekistan[MeSH Terms])) OR (Vanuatu[MeSH Terms])) OR (Vietnam[MeSH Terms])) OR (West Bank[MeSH Terms])) OR (Yemen[MeSH Terms])) OR (Zambia[MeSH Terms])) OR (Zimbabwe[MeSH Terms])** | | |
| #3 | **(((((((((Under-five children) OR (Child)) OR (Infant)) OR (Preschool)) OR (Toddler)) OR (Childhood)) OR (Child[MeSH Terms])) OR (Infant[MeSH Terms])) OR (Child[MeSH Terms])) OR (preschool[MeSH Terms])** | 1,369,452 | February 22, 2022 02:27:50 |
| #4 | ("diarrhea"[MeSH Terms] OR "diarrhea"[All Fields] OR "diarrheas"[All Fields] OR "diarrhoea"[All Fields] OR "diarrhoeas"[All Fields] OR ("diarrhea"[MeSH Terms] OR "diarrhea"[All Fields] OR "diarrheas"[All Fields] OR "diarrhoea"[All Fields] OR "diarrhoeas"[All Fields]) OR ("dysentery"[MeSH Terms] OR "dysentery"[All Fields] OR "dysenteries"[All Fields]) OR (("acute"[All Fields] OR "acutely"[All Fields] OR "acutes"[All Fields]) AND ("diarrheal"[All Fields] OR "diarrheals"[All Fields] OR "diarrhoeal"[All Fields] OR "diarrhoeals"[All Fields]) AND ("disease"[MeSH Terms] OR "disease"[All Fields] OR "diseases"[All Fields] OR "disease s"[All Fields] OR "diseased"[All Fields])) OR ("epidemiology"[MeSH Subheading] OR "epidemiology"[All Fields] OR "prevalence"[All Fields] OR "prevalence"[MeSH Terms] OR "prevalance"[All Fields] OR "prevalences"[All Fields] OR "prevalence s"[All Fields] OR "prevalent"[All Fields] OR "prevalently"[All Fields] OR "prevalents"[All Fields]) OR ("epidemiology"[MeSH Subheading] OR "epidemiology"[All Fields] OR "incidence"[All Fields] OR "incidence"[MeSH Terms] OR "incidences"[All Fields] OR "incident"[All Fields] OR "incidents"[All Fields]) OR ("cohort studies"[MeSH Terms] OR ("cohort"[All Fields] AND "studies"[All Fields]) OR "cohort studies"[All Fields] OR ("incidence"[All Fields] AND "study"[All Fields]) OR "incidence study"[All Fields]) OR ("incidence"[MeSH Terms] OR "incidence"[All Fields] OR ("incidence"[All Fields] AND "rate"[All Fields]) OR "incidence rate"[All Fields]) OR ("incidence"[MeSH Terms] OR "incidence"[All Fields] OR ("cumulative"[All Fields] AND "incidence"[All Fields]) OR "cumulative incidence"[All Fields]) OR ("epidemiology"[MeSH Subheading] OR "epidemiology"[All Fields] OR "frequency"[All Fields] OR "epidemiology"[MeSH Terms] OR "frequence"[All Fields] OR "frequences"[All Fields] OR "frequencies"[All Fields]) OR ("epidemiology"[MeSH Subheading] OR "epidemiology"[All Fields] OR "surveillance"[All Fields] OR "epidemiology"[MeSH Terms] OR "surveilance"[All Fields] OR "surveillances"[All Fields] OR "surveilled"[All Fields] OR "surveillence"[All Fields]) OR ("burdon"[All Fields] AND ("disease"[MeSH Terms] OR "disease"[All Fields] OR "diseases"[All Fields] OR "disease s"[All Fields] OR "diseased"[All Fields])) OR (("acute"[All Fields] OR "acutely"[All Fields] | 229,175 | February 22, 2022 02:30:25 |

| | | | |
|---|---|---|---|
| | OR "acutes"[All Fields]) AND ("diarrhea"[MeSH Terms] OR "diarrhea"[All Fields] OR "diarrheas"[All Fields] OR "diarrhoea"[All Fields] OR "diarrhoeas"[All Fields])) OR (("acute"[All Fields] OR "acutely"[All Fields] OR "acutes"[All Fields]) AND ("diarrheal"[All Fields] OR "diarrheals"[All Fields] OR "diarrhoeal"[All Fields] OR "diarrhoeals"[All Fields]) AND ("disease"[MeSH Terms] OR "disease"[All Fields] OR "diseases"[All Fields] OR "disease s"[All Fields] OR "diseased"[All Fields]) AND ("child"[MeSH Terms] OR "child"[All Fields] OR "children"[All Fields] OR "child s"[All Fields] OR "children s"[All Fields] OR "childrens"[All Fields] OR "childs"[All Fields])) OR "diarrhea"[MeSH Terms] OR "diarrhea/epidemiology"[MeSH Terms] OR "epidemiology"[MeSH Terms] OR "public health surveillance"[MeSH Terms] OR "population surveillance"[MeSH Terms] OR "epidemiological monitoring"[MeSH Terms] OR "diarrhea, infantile"[MeSH Terms] OR "gastroenteritis"[MeSH Terms] OR "dysentery"[MeSH Terms] OR "gastrointestinal diseases"[MeSH Terms]) AND ((("poverty"[MeSH Terms] OR "poverty"[All Fields] OR ("low"[All Fields] AND "income"[All Fields]) OR "low income"[All Fields]) AND ("countries"[All Fields] OR "country"[All Fields] OR "country s"[All Fields] OR "countrys"[All Fields])) OR (("middle"[All Fields] OR "middles"[All Fields]) AND ("income"[MeSH Terms] OR "income"[All Fields] OR "incomes"[All Fields] OR "income s"[All Fields]) AND ("countries"[All Fields] OR "country"[All Fields] OR "country s"[All Fields] OR "countrys"[All Fields])) OR ("developing countries"[MeSH Terms] OR ("developing"[All Fields] AND "countries"[All Fields]) OR "developing countries"[All Fields]) OR ("developing countries"[MeSH Terms] OR ("developing"[All Fields] AND "countries"[All Fields]) OR "developing countries"[All Fields] OR ("least"[All Fields] AND "developed"[All Fields] AND "countries"[All Fields]) OR "least developed countries"[All Fields]) OR ("developing countries"[MeSH Terms] OR ("developing"[All Fields] AND "countries"[All Fields]) OR "developing countries"[All Fields] OR ("less"[All Fields] AND "developed"[All Fields] AND "countries"[All Fields]) OR "less developed countries"[All Fields]) OR ("developing countries"[MeSH Terms] OR ("developing"[All Fields] AND "countries"[All Fields]) OR "developing countries"[All Fields] OR ("third"[All Fields] AND "world"[All Fields] AND "countries"[All Fields]) OR "third world countries"[All Fields]) OR (("poverty"[MeSH Terms] OR "poverty"[All Fields] OR ("low"[All Fields] AND "income"[All Fields]) OR "low income"[All Fields]) AND ("countries"[All Fields] OR "country"[All Fields] OR "country s"[All Fields] OR "countrys"[All Fields])) OR ("central african republic"[MeSH Terms] OR ("central"[All Fields] AND "african"[All Fields] AND "republic"[All Fields]) OR "central african republic"[All Fields]) OR ("yemen"[MeSH Terms] OR "yemen"[All Fields]) OR ("ethiopia"[MeSH Terms] OR "ethiopia"[All Fields] OR "ethiopia s"[All Fields]) OR ("niger"[MeSH Terms] OR "niger"[All Fields]) OR ("gambia"[MeSH Terms] OR "gambia"[All Fields] OR "gambia s"[All Fields]) OR ("rwanda"[MeSH Terms] OR | | |

| | | | |
|---|---|---|---|
| | "rwanda"[All Fields] OR "rwanda s"[All Fields]) OR ("guinea"[MeSH Terms] OR "guinea"[All Fields] OR "guinea s"[All Fields] OR "guineas"[All Fields]) OR ("sierra leone"[MeSH Terms] OR ("sierra"[All Fields] AND "leone"[All Fields]) OR "sierra leone"[All Fields]) OR ("angola"[MeSH Terms] OR "angola"[All Fields] OR "angola s"[All Fields]) OR ("honduras"[MeSH Terms] OR "honduras"[All Fields]) OR ("philippine"[All Fields] OR "philippines"[MeSH Terms] OR "philippines"[All Fields]) OR ("algeria"[MeSH Terms] OR "algeria"[All Fields]) OR ("india"[MeSH Terms] OR "india"[All Fields] OR "india s"[All Fields] OR "indias"[All Fields]) OR ("samoa"[MeSH Terms] OR "samoa"[All Fields] OR "samoas"[All Fields]) OR ("bangladesh"[MeSH Terms] OR "bangladesh"[All Fields] OR "bangladesh s"[All Fields]) OR ("indonesia"[MeSH Terms] OR "indonesia"[All Fields] OR "indonesia s"[All Fields] OR "indonesias"[All Fields]) OR ("sao"[All Fields] AND "tome"[All Fields]) OR ("principe"[All Fields] OR "principes"[All Fields]) OR ("belize"[MeSH Terms] OR "belize"[All Fields]) OR ("iran"[MeSH Terms] OR "iran"[All Fields]) OR ("senegal"[MeSH Terms] OR "senegal"[All Fields] OR "senegal s"[All Fields]) OR ("benin"[MeSH Terms] OR "benin"[All Fields] OR "benin s"[All Fields]) OR ("kenya"[MeSH Terms] OR "kenya"[All Fields] OR "kenya s"[All Fields]) OR ("melanesia"[MeSH Terms] OR "melanesia"[All Fields] OR ("solomon"[All Fields] AND "islands"[All Fields]) OR "solomon islands"[All Fields]) OR ("bhutan"[MeSH Terms] OR "bhutan"[All Fields] OR "bhutan s"[All Fields]) OR ("micronesia"[MeSH Terms] OR "micronesia"[All Fields] OR "kiribati"[All Fields]) OR ("sri lanka"[MeSH Terms] OR ("sri"[All Fields] AND "lanka"[All Fields]) OR "sri lanka"[All Fields]) OR ("bolivia"[MeSH Terms] OR "bolivia"[All Fields]) OR ("kyrgyzstan"[MeSH Terms] OR "kyrgyzstan"[All Fields] OR ("kyrgyz"[All Fields] AND "republic"[All Fields]) OR "kyrgyz republic"[All Fields]) OR ("lao"[All Fields] AND "pdr"[All Fields]) OR ("tajikistan"[MeSH Terms] OR "tajikistan"[All Fields]) OR ("cambodia"[MeSH Terms] OR "cambodia"[All Fields] OR "cambodia s"[All Fields]) OR ("lesotho"[MeSH Terms] OR "lesotho"[All Fields]) OR ("timor leste"[MeSH Terms] OR "timor leste"[All Fields] OR ("timor"[All Fields] AND "leste"[All Fields]) OR "timor leste"[All Fields]) OR ("cameroon"[MeSH Terms] OR "cameroon"[All Fields] OR "cameroons"[All Fields] OR "cameroon s"[All Fields]) OR ("mauritania"[MeSH Terms] OR "mauritania"[All Fields]) OR ("tunisia"[MeSH Terms] OR "tunisia"[All Fields]) OR ("comoros"[MeSH Terms] OR "comoros"[All Fields] OR "comoro"[All Fields]) OR ("micronesia"[MeSH Terms] OR "micronesia"[All Fields]) OR ("ukraine"[MeSH Terms] OR "ukraine"[All Fields] OR "ukraine s"[All Fields]) OR ("congo"[MeSH Terms] OR "congo"[All Fields]) OR ("mongolia"[MeSH Terms] OR "mongolia"[All Fields] OR "mongolia s"[All Fields]) OR ("uzbekistan"[MeSH Terms] OR "uzbekistan"[All Fields]) OR ("cote d ivoire"[MeSH Terms] OR ("cote"[All Fields] AND "d ivoire"[All Fields]) OR "cote d ivoire"[All Fields]) OR ("morocco"[MeSH Terms] OR "morocco"[All Fields]) OR ("vanuatu"[MeSH Terms] OR "vanuatu"[All Fields]) OR | | |

| | | | |
|---|---|---|---|
| | ("djibouti"[MeSH Terms] OR "djibouti"[All Fields]) OR ("myanmar"[MeSH Terms] OR "myanmar"[All Fields] OR "myanmar s"[All Fields] OR "myanmars"[All Fields]) OR ("vietnam"[MeSH Terms] OR "vietnam"[All Fields] OR "vietnam s"[All Fields]) OR ("egypt"[MeSH Terms] OR "egypt"[All Fields] OR "egypt s"[All Fields]) OR ("nepal"[MeSH Terms] OR "nepal"[All Fields] OR "nepal s"[All Fields]) OR ("middle east"[MeSH Terms] OR ("middle"[All Fields] AND "east"[All Fields]) OR "middle east"[All Fields] OR ("west"[All Fields] AND "bank"[All Fields]) OR "west bank"[All Fields]) OR "gaza"[All Fields] OR ("brazil"[MeSH Terms] OR "brazil"[All Fields] OR "brazil s"[All Fields] OR "brazils"[All Fields]) OR ("kazakhstan"[MeSH Terms] OR "kazakhstan"[All Fields] OR "kazakhstan s"[All Fields]) OR ("south africa"[MeSH Terms] OR ("south"[All Fields] AND "africa"[All Fields]) OR "south africa"[All Fields]) OR ("bulgaria"[MeSH Terms] OR "bulgaria"[All Fields]) OR ("kosovo"[MeSH Terms] OR "kosovo"[All Fields] OR "kosovo s"[All Fields]) OR ("saint lucia"[MeSH Terms] OR ("saint"[All Fields] AND "lucia"[All Fields]) OR "saint lucia"[All Fields] OR ("st"[All Fields] AND "lucia"[All Fields]) OR "st lucia"[All Fields]) OR ("china"[MeSH Terms] OR "china"[All Fields] OR "china s"[All Fields] OR "chinas"[All Fields]) OR ("lebanon"[MeSH Terms] OR "lebanon"[All Fields] OR "lebanon s"[All Fields]) OR (vincent, st[Investigator] OR st vincent[Author] OR st vincent[Investigator]) OR ("saint vincent and the grenadines"[MeSH Terms] OR ("saint"[All Fields] AND "vincent"[All Fields] AND "grenadines"[All Fields]) OR "saint vincent and the grenadines"[All Fields] OR "grenadines"[All Fields]) OR ("colombia"[MeSH Terms] OR "colombia"[All Fields] OR "colombia s"[All Fields]) OR ("libya"[MeSH Terms] OR "libya"[All Fields]) OR ("suriname"[MeSH Terms] OR "suriname"[All Fields] OR "surinam"[All Fields]) OR ("costa rica"[MeSH Terms] OR ("costa"[All Fields] AND "rica"[All Fields]) OR "costa rica"[All Fields]) OR ("malaysia"[MeSH Terms] OR "malaysia"[All Fields] OR "malaysia s"[All Fields]) OR ("dominican republic"[MeSH Terms] OR ("dominican"[All Fields] AND "republic"[All Fields]) OR "dominican republic"[All Fields]) OR ("mauritius"[MeSH Terms] OR "mauritius"[All Fields]) OR ("turkmenistan"[MeSH Terms] OR "turkmenistan"[All Fields]) OR ("equatorial guinea"[MeSH Terms] OR ("equatorial"[All Fields] AND "guinea"[All Fields]) OR "equatorial guinea"[All Fields]) OR ("mexico"[MeSH Terms] OR "mexico"[All Fields] OR "mexico s"[All Fields] OR "mexicos"[All Fields]) OR ("micronesia"[MeSH Terms] OR "micronesia"[All Fields] OR "tuvalu"[All Fields]) OR ("ecuador"[MeSH Terms] OR "ecuador"[All Fields] OR "ecuador s"[All Fields]) OR ("moldova"[MeSH Terms] OR "moldova"[All Fields]) OR ("fiji"[MeSH Terms] OR "fiji"[All Fields]) OR ("montenegro"[MeSH Terms] OR "montenegro"[All Fields]) OR ("afghanistan"[MeSH Terms] OR "afghanistan"[All Fields] OR "afghanistan s"[All Fields]) OR ("guinea bissau"[MeSH Terms] OR "guinea bissau"[All Fields] OR ("guinea"[All Fields] AND "bissau"[All Fields]) OR "guinea bissau"[All Fields]) OR ("somalia"[MeSH | | |

| | | | |
|---|---|---|---|
| | Terms] OR "somalia"[All Fields]) OR ("burkina faso"[MeSH Terms] OR ("burkina"[All Fields] AND "faso"[All Fields]) OR "burkina faso"[All Fields]) OR ("korea"[MeSH Terms] OR "korea"[All Fields] OR "korea s"[All Fields] OR "koreas"[All Fields]) OR ("south sudan"[MeSH Terms] OR ("south"[All Fields] AND "sudan"[All Fields]) OR "south sudan"[All Fields]) OR ("burundi"[MeSH Terms] OR "burundi"[All Fields]) OR ("liberia"[MeSH Terms] OR "liberia"[All Fields] OR "liberia s"[All Fields]) OR ("sudan"[MeSH Terms] OR "sudan"[All Fields] OR "sudans"[All Fields] OR "sudan s"[All Fields]) OR ("madagascar"[MeSH Terms] OR "madagascar"[All Fields] OR "madagascar s"[All Fields]) OR ("syria"[MeSH Terms] OR "syria"[All Fields] OR "syria s"[All Fields]) OR ("chad"[MeSH Terms] OR "chad"[All Fields]) OR ("malawi"[MeSH Terms] OR "malawi"[All Fields] OR "malawi s"[All Fields]) OR ("togo"[MeSH Terms] OR "togo"[All Fields]) OR ("congo"[MeSH Terms] OR "congo"[All Fields]) OR ("dem"[All Fields] AND "rep"[All Fields]) OR ("mali"[MeSH Terms] OR "mali"[All Fields]) OR ("uganda"[MeSH Terms] OR "uganda"[All Fields] OR "uganda s"[All Fields]) OR ("eritrea"[MeSH Terms] OR "eritrea"[All Fields]) OR ("mozambique"[MeSH Terms] OR "mozambique"[All Fields] OR "mozambique s"[All Fields]) OR ("ethiopia"[MeSH Terms] OR "ethiopia"[All Fields] OR "ethiopia s"[All Fields]) OR ("niger"[MeSH Terms] OR "niger"[All Fields]) OR ("gambia"[MeSH Terms] OR "gambia"[All Fields] OR "gambia s"[All Fields]) OR ("rwanda"[MeSH Terms] OR "rwanda"[All Fields] OR "rwanda s"[All Fields]) OR (("guinea"[MeSH Terms] OR "guinea"[All Fields] OR "guinea s"[All Fields] OR "guineas"[All Fields]) AND ("sierra leone"[MeSH Terms] OR ("sierra"[All Fields] AND "leone"[All Fields]) OR "sierra leone"[All Fields])) OR ("angola"[MeSH Terms] OR "angola"[All Fields] OR "angola s"[All Fields]) OR ("honduras"[MeSH Terms] OR "honduras"[All Fields]) OR ("philippine"[All Fields] OR "philippines"[MeSH Terms] OR "philippines"[All Fields]) OR ("algeria"[MeSH Terms] OR "algeria"[All Fields]) OR ("india"[MeSH Terms] OR "india"[All Fields] OR "india s"[All Fields] OR "indias"[All Fields]) OR ("samoa"[MeSH Terms] OR "samoa"[All Fields] OR "samoas"[All Fields]) OR ("bangladesh"[MeSH Terms] OR "bangladesh"[All Fields] OR "bangladesh s"[All Fields]) OR ("indonesia"[MeSH Terms] OR "indonesia"[All Fields] OR "indonesia s"[All Fields] OR "indonesias"[All Fields]) OR ("sao"[All Fields] AND "tome"[All Fields]) OR ("principe"[All Fields] OR "principes"[All Fields]) OR ("tanzania"[MeSH Terms] OR "tanzania"[All Fields] OR "tanzania s"[All Fields]) OR ("cabo verde"[MeSH Terms] OR ("cabo"[All Fields] AND "verde"[All Fields]) OR "cabo verde"[All Fields]) OR ("eswatini"[MeSH Terms] OR "eswatini"[All Fields]) OR ("nigeria"[MeSH Terms] OR "nigeria"[All Fields] OR "nigeria s"[All Fields]) OR ("zimbabwe"[MeSH Terms] OR "zimbabwe"[All Fields] OR "zimbabwe s"[All Fields]) OR ("ghana"[MeSH Terms] OR "ghana"[All Fields] OR "ghana s"[All Fields]) OR ("pakistan"[MeSH Terms] OR "pakistan"[All Fields] OR "pakistan s"[All Fields]) OR ("haiti"[MeSH Terms] OR | | |

| | | | |
|---|---|---|---|
| | "haiti"[All Fields] OR "haiti s"[All Fields]) OR ("papua new guinea"[MeSH Terms] OR ("papua"[All Fields] AND "new"[All Fields] AND "guinea"[All Fields]) OR "papua new guinea"[All Fields]) OR ("albania"[MeSH Terms] OR "albania"[All Fields]) OR ("namibia"[MeSH Terms] OR "namibia"[All Fields]) OR ("american samoa"[MeSH Terms] OR ("american"[All Fields] AND "samoa"[All Fields]) OR "american samoa"[All Fields]) OR ("georgia"[MeSH Terms] OR "georgia"[All Fields] OR "georgia republic"[MeSH Terms] OR ("georgia"[All Fields] AND "republic"[All Fields]) OR "georgia republic"[All Fields] OR "georgia s"[All Fields]) OR ("gabon"[MeSH Terms] OR "gabon"[All Fields]) OR ("republic of north macedonia"[MeSH Terms] OR ("republic"[All Fields] AND "north"[All Fields] AND "macedonia"[All Fields]) OR "republic of north macedonia"[All Fields] OR ("north"[All Fields] AND "macedonia"[All Fields]) OR "north macedonia"[All Fields]) OR ("argentina"[MeSH Terms] OR "argentina"[All Fields] OR "argentina s"[All Fields] OR "argentinae"[All Fields]) OR ("grenada"[MeSH Terms] OR "grenada"[All Fields]) OR ("panama"[MeSH Terms] OR "panama"[All Fields] OR "panama s"[All Fields]) OR ("armenia"[MeSH Terms] OR "armenia"[All Fields]) OR ("guatemala"[MeSH Terms] OR "guatemala"[All Fields] OR "guatemala s"[All Fields]) OR ("paraguai"[All Fields] OR "paraguay"[MeSH Terms] OR "paraguay"[All Fields]) OR ("el salvador"[MeSH Terms] OR ("el"[All Fields] AND "salvador"[All Fields]) OR "el salvador"[All Fields]) OR ("nicaragua"[MeSH Terms] OR "nicaragua"[All Fields] OR "nicaragua s"[All Fields]) OR ("zambia"[MeSH Terms] OR "zambia"[All Fields] OR "zambia s"[All Fields]) OR ("azerbaijan"[MeSH Terms] OR "azerbaijan"[All Fields]) OR ("guyana"[MeSH Terms] OR "guyana"[All Fields]) OR ("peru"[MeSH Terms] OR "peru"[All Fields]) OR ("republic of belarus"[MeSH Terms] OR ("republic"[All Fields] AND "belarus"[All Fields]) OR "republic of belarus"[All Fields] OR "belarus"[All Fields]) OR ("iraq"[MeSH Terms] OR "iraq"[All Fields]) OR ("romania"[MeSH Terms] OR "romania"[All Fields] OR "romania s"[All Fields]) OR ("bosnia and herzegovina"[MeSH Terms] OR ("bosnia"[All Fields] AND "herzegovina"[All Fields]) OR "bosnia and herzegovina"[All Fields] OR "bosnia"[All Fields]) OR ("bosnia and herzegovina"[MeSH Terms] OR ("bosnia"[All Fields] AND "herzegovina"[All Fields]) OR "bosnia and herzegovina"[All Fields] OR "herzegovina"[All Fields]) OR ("jamaica"[MeSH Terms] OR "jamaica"[All Fields] OR "jamaica s"[All Fields]) OR ("russia"[MeSH Terms] OR "russia"[All Fields] OR "russia s"[All Fields] OR "russias"[All Fields]) OR ("botswana"[MeSH Terms] OR "botswana"[All Fields] OR "botswana s"[All Fields]) OR ("jordan"[MeSH Terms] OR "jordan"[All Fields]) OR ("serbia"[MeSH Terms] OR "serbia"[All Fields]) OR ("thailand"[MeSH Terms] OR "thailand"[All Fields] OR "thailand s"[All Fields]) OR ("cuba"[MeSH Terms] OR "cuba"[All Fields]) OR ("indian ocean islands"[MeSH Terms] OR ("indian"[All Fields] AND "ocean"[All Fields] AND "islands"[All Fields]) OR "indian ocean islands"[All Fields] OR "maldives"[All Fields] OR "maldive"[All Fields]) OR | | |

| | | | |
|---|---|---|---|
| | ("tonga"[MeSH Terms] OR "tonga"[All Fields] OR "tonga s"[All Fields]) OR ("dominica"[MeSH Terms] OR "dominica"[All Fields]) OR ("micronesia"[MeSH Terms] OR "micronesia"[All Fields] OR ("marshall"[All Fields] AND "islands"[All Fields]) OR "marshall islands"[All Fields]) OR ("turkey"[MeSH Terms] OR "turkey"[All Fields] OR "turkey s"[All Fields] OR "turkeys"[MeSH Terms] OR "turkeys"[All Fields]) OR ("equatorial guinea"[MeSH Terms] OR ("equatorial"[All Fields] AND "guinea"[All Fields]) OR "equatorial guinea"[All Fields]) OR ("mexico"[MeSH Terms] OR "mexico"[All Fields] OR "mexico s"[All Fields] OR "mexicos"[All Fields]) OR (("micronesia"[MeSH Terms] OR "micronesia"[All Fields] OR "tuvalu"[All Fields]) AND ("ecuador"[MeSH Terms] OR "ecuador"[All Fields] OR "ecuador s"[All Fields])) OR "developing countries"[MeSH Terms] OR "developing countries"[MeSH Terms] OR "developing countries"[MeSH Terms] OR "developing countries"[MeSH Terms] OR "afghanistan"[MeSH Terms] OR "albania"[MeSH Terms] OR "algeria"[MeSH Terms] OR "argentina"[MeSH Terms] OR "armenia"[MeSH Terms] OR "angola"[MeSH Terms] OR "american samoa"[MeSH Terms] OR "azerbaijan"[MeSH Terms] OR "bangladesh"[MeSH Terms] OR "republic of belarus"[MeSH Terms] OR "belize"[MeSH Terms] OR "benin"[MeSH Terms] OR "bhutan"[MeSH Terms] OR "bolivia"[MeSH Terms] OR "bosnia and herzegovina"[MeSH Terms] OR "botswana"[MeSH Terms] OR "brazil"[MeSH Terms] OR "bulgaria"[MeSH Terms] OR "burkina faso"[MeSH Terms] OR "burundi"[MeSH Terms] OR "cabo verde"[MeSH Terms] OR "cambodia"[MeSH Terms] OR "cameroon"[MeSH Terms] OR "central african republic"[MeSH Terms] OR "chad"[MeSH Terms] OR "china"[MeSH Terms] OR "colombia"[MeSH Terms] OR "comoros"[MeSH Terms] OR "congo"[MeSH Terms] OR "congo"[MeSH Terms] OR "costa rica"[MeSH Terms] OR "cote d ivoire"[MeSH Terms] OR "cuba"[MeSH Terms] OR "djibouti"[MeSH Terms] OR "dominica"[MeSH Terms] OR "dominican republic"[MeSH Terms] OR "ecuador"[MeSH Terms] OR "egypt"[MeSH Terms] OR "el salvador"[MeSH Terms] OR "equatorial guinea"[MeSH Terms] OR "eritrea"[MeSH Terms] OR "eswatini"[MeSH Terms] OR "ethiopia"[MeSH Terms] OR "fiji"[MeSH Terms] OR "gabon"[MeSH Terms] OR "gambia"[MeSH Terms] OR ("georgia"[MeSH Terms] OR "georgia republic"[MeSH Terms]) OR "ghana"[MeSH Terms] OR "grenada"[MeSH Terms] OR "guatemala"[MeSH Terms] OR "guinea"[MeSH Terms] OR "guinea bissau"[MeSH Terms] OR "guyana"[MeSH Terms] OR "haiti"[MeSH Terms] OR "bosnia and herzegovina"[MeSH Terms] OR "honduras"[MeSH Terms] OR "india"[MeSH Terms] OR "indonesia"[MeSH Terms] OR "iran"[MeSH Terms] OR "iraq"[MeSH Terms] OR "jamaica"[MeSH Terms] OR "jordan"[MeSH Terms] OR "kazakhstan"[MeSH Terms] OR "kenya"[MeSH Terms] OR "micronesia"[MeSH Terms] OR "korea"[MeSH Terms] OR "kosovo"[MeSH Terms] OR "kyrgyzstan"[MeSH Terms] OR "lebanon"[MeSH Terms] OR "lesotho"[MeSH Terms] OR "liberia"[MeSH Terms] OR "libya"[MeSH Terms] OR "madagascar"[MeSH Terms] OR | | |

| | | | |
|---|---|---|---|
| | "malawi"[MeSH Terms] OR "malaysia"[MeSH Terms] OR "indian ocean islands"[MeSH Terms] OR "mali"[MeSH Terms] OR "micronesia"[MeSH Terms] OR "mauritania"[MeSH Terms] OR "mauritius"[MeSH Terms] OR "mexico"[MeSH Terms] OR "micronesia"[MeSH Terms] OR "moldova"[MeSH Terms] OR "mongolia"[MeSH Terms] OR "montenegro"[MeSH Terms] OR "morocco"[MeSH Terms] OR "mozambique"[MeSH Terms] OR "myanmar"[MeSH Terms] OR "namibia"[MeSH Terms] OR "nepal"[MeSH Terms] OR "nicaragua"[MeSH Terms] OR "niger"[MeSH Terms] OR "nigeria"[MeSH Terms] OR "republic of north macedonia"[MeSH Terms] OR "pakistan"[MeSH Terms] OR "panama"[MeSH Terms] OR "papua new guinea"[MeSH Terms] OR "paraguay"[MeSH Terms] OR "peru"[MeSH Terms] OR "philippines"[MeSH Terms] OR "romania"[MeSH Terms] OR "russia"[MeSH Terms] OR "rwanda"[MeSH Terms] OR "samoa"[MeSH Terms] OR "senegal"[MeSH Terms] OR "serbia"[MeSH Terms] OR "sierra leone"[MeSH Terms] OR "melanesia"[MeSH Terms] OR "somalia"[MeSH Terms] OR "south africa"[MeSH Terms] OR "south sudan"[MeSH Terms] OR "saint lucia"[MeSH Terms] OR "sudan"[MeSH Terms] OR "suriname"[MeSH Terms] OR "sri lanka"[MeSH Terms] OR "syria"[MeSH Terms] OR "tajikistan"[MeSH Terms] OR "tanzania"[MeSH Terms] OR "thailand"[MeSH Terms] OR ("saint vincent and the grenadines"[MeSH Terms]) OR "timor leste"[MeSH Terms] OR "togo"[MeSH Terms] OR "tonga"[MeSH Terms] OR "tunisia"[MeSH Terms] OR "turkey"[MeSH Terms] OR "turkmenistan"[MeSH Terms] OR "micronesia"[MeSH Terms] OR "uganda"[MeSH Terms] OR "ukraine"[MeSH Terms] OR "uzbekistan"[MeSH Terms] OR "vanuatu"[MeSH Terms] OR "vietnam"[MeSH Terms] OR "middle east"[MeSH Terms] OR "yemen"[MeSH Terms] OR "zambia"[MeSH Terms] OR "zimbabwe"[MeSH Terms]) AND (("under five"[All Fields] AND ("child"[MeSH Terms] OR "child"[All Fields] OR "children"[All Fields] OR "child s"[All Fields] OR "children s"[All Fields] OR "childrens"[All Fields] OR "childs"[All Fields])) OR ("child"[MeSH Terms] OR "child"[All Fields] OR "children"[All Fields] OR "child s"[All Fields] OR "children s"[All Fields] OR "childrens"[All Fields] OR "childs"[All Fields]) OR ("infant"[MeSH Terms] OR "infant"[All Fields] OR "infants"[All Fields] OR "infant s"[All Fields]) OR ("child, preschool"[MeSH Terms] OR ("child"[All Fields] AND "preschool"[All Fields]) OR "preschool child"[All Fields] OR "preschooler"[All Fields] OR "preschoolers"[All Fields] OR "preschool"[All Fields] OR "preschooler s"[All Fields] OR "preschools"[All Fields]) OR ("toddler"[All Fields] OR "toddler s"[All Fields] OR "toddlers"[All Fields]) OR ("childhood"[All Fields] OR "childhoods"[All Fields]) OR "child"[MeSH Terms] OR "infant"[MeSH Terms] OR "child"[MeSH Terms]) | | |

Logic Grid of search and MeSH terms of Embase for systematic review of incidence of diarrhea among under-five children in low- and Middle-income countries.

| **Condition** | **Context** | | **Population** |
|---|---|---|---|
| **Text words** | **Text words** | | **Text words** |
| • Diarrhea<br>• Dysentery<br>• Acute diarrheal disease<br>• Prevalence<br>• Incidence<br>• Incidence study<br>• Incidence rate<br>• Cumulative incidence<br>• Frequency<br>• Monitoring<br>• disease burden<br>• Acute diarrhea<br>• Acute Diarrheal Disease in Children | • Low-income countries<br>• Middle income countries<br>• Developing country<br>• Least Developed Countries<br>• Less Developed Countries<br>• Third-World Countries<br>• Low-Income Countries<br>• Central African Republic<br>• Afghanistan<br>• Albania<br>• Algeria<br>• American Samoa<br>• Angola<br>• Argentina<br>• Armenia<br>• Azerbaijan<br>• Bangladesh<br>• Belarus<br>• Belize<br>• Benin<br>• Bhutan<br>• Bolivia<br>• Bosnia<br>• Botswana<br>• Brazil<br>• Bulgaria<br>• Burkina Faso<br>• Burundi<br>• Cabo Verde<br>• Cambodia<br>• Cameroon<br>• Chad<br>• China<br>• Colombia<br>• Comoros<br>• Liberia | • Congo<br>• Congo<br>• Costa Rica<br>• Côte d'Ivoire<br>• Cuba<br>• Dem. Rep<br>• Djibouti<br>• Dominica<br>• Dominican Republic<br>• Ecuador<br>• Ecuador<br>• Egypt<br>• El Salvador<br>• Equatorial Guinea<br>• Equatorial Guinea<br>• Eritrea<br>• Eswatini<br>• Ethiopia<br>• Ethiopia<br>• Fiji<br>• Gabon<br>• Gambia<br>• Gambia<br>• Gaza<br>• Georgia<br>• Ghana<br>• Grenada<br>• Guatemala<br>• Guinea<br>• Guinea<br>• Guinea-Bissau<br>• Guyana<br>• Haiti<br>• Herzegovina<br>• Honduras<br>• Honduras | • Under-five children<br>• Child<br>• Infant<br>• Preschool<br>• Toddler<br>• Childhood |

<table>
<tr>
<td></td>
<td>

- Libya
- Madagascar
- Malawi
- Malaysia
- Maldives
- Mali
- Marshall Islands
- Mauritania
- Mauritius
- Mexico
- Mexico
- Micronesia
- Moldova
- Mongolia
- Montenegro
- Morocco
- Mozambique
- Myanmar
- Namibia
- Nepal
- Nicaragua
- Niger
- Niger
- Nigeria
- North Macedonia
- Pakistan
- Panama
- Papua New Guinea
- Paraguay
- Peru
- Philippines
- Philippines
- Principe
- Principe
- Romania
- Russia
- Rwanda
- Rwanda
- Samoa
- Samoa
- São Tomé
- São Tomé
- Senegal
- Yemen

</td>
<td>

- India
- India
- Indonesia
- Indonesia
- Iran
- Iraq
- Jamaica
- Jordan
- Kazakhstan
- Kenya
- Kiribati
- Korea
- Kosovo
- Kyrgyz Republic
- Lao PDR
- Lebanon
- Lesotho
- Serbia
- Sierra Leone
- Sierra Leone
- Solomon Islands
- Somalia
- South Africa
- South Sudan
- Sri Lanka
- St. Lucia
- St. Vincent
- Sudan
- Suriname
- Syria
- Tajikistan
- Tanzania
- Thailand
- The Grenadines
- Timor-Leste
- Togo
- Tonga
- Tunisia
- Turkey
- Turkmenistan
- Tuvalu
- Tuvalu
- Uganda
- Ukraine

</td>
<td></td>
</tr>
</table>

| | | | |
|---|---|---|---|
| | • Zambia<br>• Zimbabwe | • Uzbekistan<br>• Vanuatu<br>• Vietnam<br>• West Bank | |
| **MeSH terms (Emtree term - exploded)**<br><br>• Diarrhea<br>• Diarrhea epidemiology<br>• Epidemiology<br>• Public Health Surveillance<br>• Population Surveillance<br>• Epidemiological Monitoring<br>• Infantile diarrhea<br>• Gastroenteritis<br>• Dysentery<br>• Gastrointestinal Diseases | **MeSH terms**<br><br>• Low-income countries<br>• Middle income countries<br>• Developing countries<br>• Least Developed Countries<br>• Less Developed Countries<br>• Third-World Countries<br>• Low-Income Countries<br>• Afghanistan<br>• Guinea-Bissau<br>• Somalia<br>• Burkina Faso<br>• Korea<br>• South Sudan<br>• Burundi<br>• Liberia<br>• Sudan<br>• Central African Republic<br>• Madagascar<br>• Syria<br>• Chad<br>• Malawi<br>• Togo<br>• Congo<br>• Dem. Rep<br>• Mali<br>• Uganda<br>• Eritrea<br>• Mozambique<br>• Yemen<br>• Ethiopia<br>• Niger<br>• Gambia<br>• _Rwanda<br>• Guinea<br>• Sierra Leone<br>• Angola<br>• Honduras | • Tunisia<br>• Comoros<br>• Micronesia<br>• Ukraine<br>• Congo<br>• Mongolia<br>• Uzbekistan<br>• Côte d'Ivoire<br>• Morocco<br>• Vanuatu<br>• Djibouti<br>• Myanmar<br>• Vietnam<br>• Egypt<br>• Nepal<br>• West Bank<br>• Gaza<br>• El Salvador<br>• Nicaragua<br>• Zambia<br>• Eswatini<br>• Nigeria<br>• Zimbabwe<br>• Ghana<br>• Pakistan<br>• Haiti<br>• Papua New Guinea<br>• Albania<br>• Gabon<br>• Namibia<br>• American Samoa<br>• Georgia<br>• North Macedonia<br>• Argentina<br>• Grenada<br>• Panama<br>• Armenia<br>• Guatemala<br>• Paraguay | **MeSH terms**<br><br>• Child<br>• Infant<br>• Child, preschool |

| | | | |
|---|---|---|---|
| | • Philippines<br>• Algeria<br>• India<br>• Samoa<br>• Bangladesh<br>• Indonesia<br>• São Tomé<br>• Principe<br>• Belize<br>• Iran<br>• Senegal<br>• Benin<br>• Kenya<br>• Solomon Islands<br>• Bhutan<br>• Kiribati<br>• Sri Lanka<br>• Bolivia<br>• Kyrgyz Republic<br>• Tanzania<br>• Cabo Verde<br>• Lao PDR<br>• Tajikistan<br>• Cambodia<br>• Lesotho<br>• Timor-Leste<br>• Cameroon<br>• Mauritania<br>• Equatorial Guinea<br>• Mexico<br>• Tuvalu<br>• Ecuador<br>• Moldova<br>• Fiji<br>• Montenegro<br>• Dominican Republic<br>• Mauritius<br>• Turkmenistan | • Azerbaijan<br>• Guyana<br>• Peru<br>• Belarus<br>• Iraq<br>• Romania<br>• Bosnia<br>• Herzegovina<br>• Jamaica<br>• Russia<br>• Botswana<br>• Jordan<br>• Serbia<br>• Brazil<br>• Kazakhstan<br>• South Africa<br>• Bulgaria<br>• Kosovo<br>• St. Lucia<br>• China<br>• Lebanon<br>• St. Vincent<br>• The Grenadines<br>• Colombia<br>• Libya<br>• Suriname<br>• Costa Rica<br>• Malaysia<br>• Thailand<br>• Cuba<br>• Maldives<br>• Tonga<br>• Dominica<br>• Marshall Islands<br>• Turkey | |

**Search history results from** Embase for systematic review of incidence of diarrhea among under-five children in low- and Middle-income countries.

| Search # | Search terms (filter applied *From 2010/9/1 to 2022/2/21)* | Search results | Date & time |
|---|---|---|---|
| #1 | (**diarrhoea** OR **dysentery** OR (**acute** AND **diarrheal** AND **disease**) OR **prevalence** OR **incidence** OR (**incidence** AND **study**) OR **'cumulative incidence'** OR **frequency** OR **monitoring** OR **'disease burden'** OR **'acute diarrhea'** OR (**acute** AND **diarrheal** AND **disease** AND **in** AND **children**) OR **'diarrhea'**/exp OR **'diarrhea epidemiology'**/exp OR **'epidemiology'**/exp OR **'public health surveillance'**/exp OR **'population surveillance'**/exp OR **'epidemiological monitoring'**/exp OR **'infantile diarrhea'**/exp OR **'gastroenteritis'**/exp OR **'dysentery'**/exp OR **'gastrointestinal disease'**/exp) AND [01-09-2010]/sd NOT [22-02-2022]/sd AND [2010-2022]/py | 4,385,325 | February 22, 2022 02:22:49 |
| #2 | **((((((((((((((((((((((((((((((((((((((((((((((((((((((((((((((((((((((((((((((((((((((((((((((((((((((((((((((((((((((((((((((((((((((((((((((((((((((((((((((((((((((((((((((((((((((((((((((((((((((((((((((((((((((((((((((((((((((((((((((((((((((((((((((((((((((((((((((((((((((((((((((((((((((((((((((((((((((((((((((((Low-income countries) OR (Middle income countries)) OR (Developing countries)) OR (Least Developed Countries)) OR (Less Developed Countries)) OR (Third-World Countries)) OR (Low-Income Countries)) OR (Central African Republic)) OR (Yemen)) OR (Ethiopia)) OR (Niger)) OR (Gambia)) OR (Rwanda)) OR (Guinea)) OR (Sierra Leone)) OR (Angola)) OR (Honduras)) OR (Philippines)) OR (Algeria)) OR (India)) OR (Samoa)) OR (Bangladesh)) OR (Indonesia)) OR (São Tomé)) OR (Principe)) OR (Belize)) OR (Iran)) OR (Senegal)) OR (Benin)) OR (Kenya)) OR (Solomon Islands)) OR (Bhutan)) OR (Kiribati)) OR (Sri Lanka)) OR (Bolivia)) OR (Kyrgyz Republic)) OR (Lao PDR)) OR (Tajikistan)) OR (Cambodia)) OR (Lesotho)) OR (Timor-Leste)) OR (\)) OR (Cameroon)) OR (Mauritania)) OR (Tunisia)) OR (Comoros)) OR (Micronesia)) OR (Ukraine)) OR (Congo)) OR (Mongolia)) OR (Uzbekistan)) OR (Côte d'Ivoire)) OR (Morocco)) OR (Vanuatu)) OR (Djibouti)) OR (Myanmar)) OR (Vietnam)) OR (Egypt)) OR (Nepal)) OR (West Bank)) OR (Gaza)) OR (Brazil)) OR (Kazakhstan)) OR (South Africa)) OR (Bulgaria)) OR (Kosovo)) OR (St. Lucia)) OR (China)) OR (Lebanon)) OR (St. Vincent)) OR (The Grenadines)) OR (Colombia)) OR (Libya)) OR (Suriname)) OR (Costa Rica)) OR (Malaysia)) OR (Dominican Republic)) OR (Mauritius)) OR (Turkmenistan)) OR (Equatorial Guinea)) OR (Mexico)) OR (Tuvalu)) OR (Ecuador)) OR (Moldova)) OR (Fiji)) OR (Montenegro)) OR (Afghanistan)) OR (Guinea-Bissau)) OR (Somalia)) OR (Burkina** | 4,514,596 | February 22, 2022 02:25:45 |

| | | | |
|---|---|---|---|
| | Faso)) OR (Korea)) OR (South Sudan)) OR (Burundi)) OR (Liberia)) OR (Sudan)) OR (Madagascar)) OR (Syria)) OR (Chad)) OR (Malawi)) OR (Togo)) OR (Congo)) OR (Dem. Rep)) OR (Mali)) OR (Uganda)) OR (Eritrea)) OR (Mozambique)) OR (Ethiopia)) OR (Niger)) OR (Gambia)) OR (Rwanda)) OR (Guinea Sierra Leone)) OR (Angola)) OR (Honduras)) OR (Philippines)) OR (Algeria)) OR (India)) OR (Samoa)) OR (Bangladesh)) OR (Indonesia)) OR (São Tomé)) OR (Principe)) OR (Tanzania)) OR (Cabo Verde)) OR (Eswatini)) OR (Nigeria)) OR (Zimbabwe)) OR (Ghana)) OR (Pakistan)) OR (Haiti)) OR (Papua New Guinea)) OR (Albania)) OR (Namibia)) OR (American Samoa)) OR (Georgia)) OR (Gabon)) OR (North Macedonia)) OR (Argentina)) OR (Grenada)) OR (Panama)) OR (Armenia)) OR (Guatemala)) OR (Paraguay)) OR (El Salvador)) OR (Nicaragua)) OR (Zambia)) OR (Azerbaijan)) OR (Guyana)) OR (Peru)) OR (Belarus)) OR (Iraq)) OR (Romania)) OR (Bosnia)) OR (Herzegovina)) OR (Jamaica)) OR (Russia)) OR (Botswana)) OR (Jordan)) OR (Serbia)) OR (Thailand)) OR (Cuba)) OR (Maldives)) OR (Tonga)) OR (Dominica)) OR (Marshall Islands)) OR (Turkey)) OR (Equatorial Guinea)) OR (Mexico)) OR (Tuvalu Ecuador)) OR (Low-income countries[MeSH Terms])) OR (Middle income countries[MeSH Terms])) OR (Developing countries[MeSH Terms])) OR (Least Developed Countries[MeSH Terms])) OR (Less Developed Countries[MeSH Terms])) OR (Third-World Countries[MeSH Terms])) OR (Low-Income Countries[MeSH Terms])) OR (Afghanistan[MeSH Terms])) OR (Albania[MeSH Terms])) OR (Algeria[MeSH Terms])) OR (Argentina[MeSH Terms])) OR (Armenia[MeSH Terms])) OR (Angola[MeSH Terms])) OR (American Samoa[MeSH Terms])) OR (Azerbaijan[MeSH Terms])) OR (Bangladesh[MeSH Terms])) OR (Belarus[MeSH Terms])) OR (Belize[MeSH Terms])) OR (Benin[MeSH Terms])) OR (Bhutan[MeSH Terms])) OR (Bolivia[MeSH Terms])) OR (Bosnia[MeSH Terms])) OR (Botswana[MeSH Terms])) OR (Brazil[MeSH Terms])) OR (Bulgaria[MeSH Terms])) OR (Burkina Faso[MeSH Terms])) OR (Burundi[MeSH Terms])) OR (Cabo Verde[MeSH Terms])) OR (Cambodia[MeSH Terms])) OR (Cameroon[MeSH Terms])) OR (Central African Republic[MeSH Terms])) OR (Chad[MeSH Terms])) OR (China[MeSH Terms])) OR (Colombia[MeSH Terms])) OR (Comoros[MeSH Terms])) OR (Congo[MeSH Terms])) OR (Congo[MeSH Terms])) OR (Costa Rica[MeSH Terms])) OR (Côte d'Ivoire[MeSH Terms])) OR (Cuba[MeSH Terms])) OR (Dem. Rep[MeSH Terms])) OR (Djibouti[MeSH Terms])) OR (Dominica[MeSH Terms])) OR (Dominican Republic[MeSH Terms])) OR (Ecuador[MeSH Terms])) OR (Egypt[MeSH Terms])) OR (El Salvador[MeSH Terms])) OR (Equatorial Guinea[MeSH Terms])) OR (Eritrea[MeSH Terms])) OR (Eswatini[MeSH Terms])) OR (Ethiopia[MeSH Terms])) OR (Fiji[MeSH Terms])) OR (Gabon[MeSH Terms])) OR (Gambia[MeSH | | |

| | | | |
|---|---|---|---|
| | **Terms])) OR (Gaza[MeSH Terms])) OR (Georgia[MeSH Terms])) OR (Ghana[MeSH Terms])) OR (Grenada[MeSH Terms])) OR (Guatemala[MeSH Terms])) OR (Guinea[MeSH Terms])) OR (Guinea-Bissau[MeSH Terms])) OR (Guyana[MeSH Terms])) OR (Haiti[MeSH Terms])) OR (Herzegovina[MeSH Terms])) OR (Honduras[MeSH Terms])) OR (India[MeSH Terms])) OR (Indonesia[MeSH Terms])) OR (Iran[MeSH Terms])) OR (Iraq[MeSH Terms])) OR (Jamaica[MeSH Terms])) OR (Jordan[MeSH Terms])) OR (Kazakhstan[MeSH Terms])) OR (Kenya[MeSH Terms])) OR (Kiribati[MeSH Terms])) OR (Korea[MeSH Terms])) OR (Kosovo[MeSH Terms])) OR (Kyrgyz Republic[MeSH Terms])) OR (Lao PDR[MeSH Terms])) OR (Lebanon[MeSH Terms])) OR (Lesotho[MeSH Terms])) OR (Liberia[MeSH Terms])) OR (Libya[MeSH Terms])) OR (Madagascar[MeSH Terms])) OR (Malawi[MeSH Terms])) OR (Malaysia[MeSH Terms])) OR (Maldives[MeSH Terms])) OR (Mali[MeSH Terms])) OR (Marshall Islands[MeSH Terms])) OR (Mauritania[MeSH Terms])) OR (Mauritius[MeSH Terms])) OR (Mexico[MeSH Terms])) OR (Micronesia[MeSH Terms])) OR (Moldova[MeSH Terms])) OR (Mongolia[MeSH Terms])) OR (Montenegro[MeSH Terms])) OR (morocco[MeSH Terms])) OR (Mozambique[MeSH Terms])) OR (Myanmar[MeSH Terms])) OR (Namibia[MeSH Terms])) OR (Nepal[MeSH Terms])) OR (Nicaragua[MeSH Terms])) OR (Niger[MeSH Terms])) OR (Nigeria[MeSH Terms])) OR (North Macedonia[MeSH Terms])) OR (Pakistan[MeSH Terms])) OR (Panama[MeSH Terms])) OR (Papua New Guinea[MeSH Terms])) OR (Paraguay[MeSH Terms])) OR (Peru[MeSH Terms])) OR (Philippines[MeSH Terms])) OR (Principe[MeSH Terms])) OR (Romania[MeSH Terms])) OR (Russia[MeSH Terms])) OR (Rwanda[MeSH Terms])) OR (Samoa[MeSH Terms])) OR (São Tomé[MeSH Terms])) OR (Senegal[MeSH Terms])) OR (Serbia[MeSH Terms])) OR (Sierra Leone[MeSH Terms])) OR (Solomon Islands[MeSH Terms])) OR (Somalia[MeSH Terms])) OR (South Africa[MeSH Terms])) OR (South Sudan[MeSH Terms])) OR (St. Lucia[MeSH Terms])) OR (St. Vincent[MeSH Terms])) OR (Sudan[MeSH Terms])) OR (Suriname[MeSH Terms])) OR (Sri Lanka[MeSH Terms])) OR (Syria[MeSH Terms])) OR (Tajikistan[MeSH Terms])) OR (Tanzania[MeSH Terms])) OR (Thailand[MeSH Terms])) OR (The Grenadines[MeSH Terms])) OR (Timor-Leste[MeSH Terms])) OR (Togo[MeSH Terms])) OR (Tonga[MeSH Terms])) OR (Tunisia[MeSH Terms])) OR (Turkey[MeSH Terms])) OR (Turkmenistan[MeSH Terms])) OR (Tuvalu[MeSH Terms])) OR (Uganda[MeSH Terms])) OR (Ukraine[MeSH Terms])) OR (Uzbekistan[MeSH Terms])) OR (Vanuatu[MeSH Terms])) OR (Vietnam[MeSH Terms])) OR (West Bank[MeSH Terms])) OR (Yemen[MeSH Terms])) OR (Zambia[MeSH Terms])) OR (Zimbabwe[MeSH Terms])** | | |

| | | | |
|---|---|---|---|
| #3 | **((((((((Under-five children) OR (Child)) OR (Infant)) OR (Preschool)) OR (Toddler)) OR (Childhood)) OR (Child[MeSH Terms])) OR (Infant[MeSH Terms])) OR (Child[MeSH Terms])) OR (preschool[MeSH Terms])** | 1,369,452 | February 22, 2022 02:27:50 |
| #4 | ("diarrhea"[MeSH Terms] OR "diarrhea"[All Fields] OR "diarrheas"[All Fields] OR "diarrhoea"[All Fields] OR "diarrhoeas"[All Fields] OR ("diarrhea"[MeSH Terms] OR "diarrhea"[All Fields] OR "diarrheas"[All Fields] OR "diarrhoea"[All Fields] OR "diarrhoeas"[All Fields]) OR ("dysentery"[MeSH Terms] OR "dysentery"[All Fields] OR "dysenteries"[All Fields]) OR (("acute"[All Fields] OR "acutely"[All Fields] OR "acutes"[All Fields]) AND ("diarrheal"[All Fields] OR "diarrheals"[All Fields] OR "diarrhoeal"[All Fields] OR "diarrhoeals"[All Fields]) AND ("disease"[MeSH Terms] OR "disease"[All Fields] OR "diseases"[All Fields] OR "disease s"[All Fields] OR "diseased"[All Fields])) OR ("epidemiology"[MeSH Subheading] OR "epidemiology"[All Fields] OR "prevalence"[All Fields] OR "prevalence"[MeSH Terms] OR "prevalance"[All Fields] OR "prevalences"[All Fields] OR "prevalence s"[All Fields] OR "prevalent"[All Fields] OR "prevalently"[All Fields] OR "prevalents"[All Fields]) OR ("epidemiology"[MeSH Subheading] OR "epidemiology"[All Fields] OR "incidence"[All Fields] OR "incidence"[MeSH Terms] OR "incidences"[All Fields] OR "incident"[All Fields] OR "incidents"[All Fields]) OR ("cohort studies"[MeSH Terms] OR ("cohort"[All Fields] AND "studies"[All Fields]) OR "cohort studies"[All Fields] OR ("incidence"[All Fields] AND "study"[All Fields]) OR "incidence study"[All Fields]) OR ("incidence"[MeSH Terms] OR "incidence"[All Fields] OR ("incidence"[All Fields] AND "rate"[All Fields]) OR "incidence rate"[All Fields]) OR ("incidence"[MeSH Terms] OR "incidence"[All Fields] OR ("cumulative"[All Fields] AND "incidence"[All Fields]) OR "cumulative incidence"[All Fields]) OR ("epidemiology"[MeSH Subheading] OR "epidemiology"[All Fields] OR "frequency"[All Fields] OR "epidemiology"[MeSH Terms] OR "frequence"[All Fields] OR "frequences"[All Fields] OR "frequencies"[All Fields]) OR ("epidemiology"[MeSH Subheading] OR "epidemiology"[All Fields] OR "surveillance"[All Fields] OR "epidemiology"[MeSH Terms] OR "surveilance"[All Fields] OR "surveillances"[All Fields] OR "surveilled"[All Fields] OR "surveillence"[All Fields]) OR ("burdon"[All Fields] AND ("disease"[MeSH Terms] OR "disease"[All Fields] OR "diseases"[All Fields] OR "disease s"[All Fields] OR "diseased"[All Fields])) OR (("acute"[All Fields] OR "acutely"[All Fields] OR "acutes"[All Fields]) AND ("diarrhea"[MeSH Terms] OR "diarrhea"[All Fields] OR "diarrheas"[All Fields] OR "diarrhoea"[All Fields] OR "diarrhoeas"[All Fields])) OR (("acute"[All Fields] OR "acutely"[All Fields] OR "acutes"[All Fields]) AND ("diarrheal"[All Fields] OR "diarrheals"[All Fields] OR "diarrhoeal"[All Fields] OR "diarrhoeals"[All Fields]) AND | 229,175 | February 22, 2022<br><br>02:30:25 |

| | | | |
|---|---|---|---|
| | ("disease"[MeSH Terms] OR "disease"[All Fields] OR "diseases"[All Fields] OR "disease s"[All Fields] OR "diseased"[All Fields]) AND ("child"[MeSH Terms] OR "child"[All Fields] OR "children"[All Fields] OR "child s"[All Fields] OR "children s"[All Fields] OR "childrens"[All Fields] OR "childs"[All Fields])) OR "diarrhea"[MeSH Terms] OR "diarrhea/epidemiology"[MeSH Terms] OR "epidemiology"[MeSH Terms] OR "public health surveillance"[MeSH Terms] OR "population surveillance"[MeSH Terms] OR "epidemiological monitoring"[MeSH Terms] OR "diarrhea, infantile"[MeSH Terms] OR "gastroenteritis"[MeSH Terms] OR "dysentery"[MeSH Terms] OR "gastrointestinal diseases"[MeSH Terms]) AND ((("poverty"[MeSH Terms] OR "poverty"[All Fields] OR ("low"[All Fields] AND "income"[All Fields]) OR "low income"[All Fields]) AND ("countries"[All Fields] OR "country"[All Fields] OR "country s"[All Fields] OR "countrys"[All Fields])) OR (("middle"[All Fields] OR "middles"[All Fields]) AND ("income"[MeSH Terms] OR "income"[All Fields] OR "incomes"[All Fields] OR "income s"[All Fields]) AND ("countries"[All Fields] OR "country"[All Fields] OR "country s"[All Fields] OR "countrys"[All Fields])) OR ("developing countries"[MeSH Terms] OR ("developing"[All Fields] AND "countries"[All Fields]) OR "developing countries"[All Fields]) OR ("developing countries"[MeSH Terms] OR ("developing"[All Fields] AND "countries"[All Fields]) OR "developing countries"[All Fields] OR ("least"[All Fields] AND "developed"[All Fields] AND "countries"[All Fields]) OR "least developed countries"[All Fields]) OR ("developing countries"[MeSH Terms] OR ("developing"[All Fields] AND "countries"[All Fields]) OR "developing countries"[All Fields] OR ("less"[All Fields] AND "developed"[All Fields] AND "countries"[All Fields]) OR "less developed countries"[All Fields]) OR ("developing countries"[MeSH Terms] OR ("developing"[All Fields] AND "countries"[All Fields]) OR "developing countries"[All Fields] OR ("third"[All Fields] AND "world"[All Fields] AND "countries"[All Fields]) OR "third world countries"[All Fields]) OR (("poverty"[MeSH Terms] OR "poverty"[All Fields] OR ("low"[All Fields] AND "income"[All Fields]) OR "low income"[All Fields]) AND ("countries"[All Fields] OR "country"[All Fields] OR "country s"[All Fields] OR "countrys"[All Fields])) OR ("central african republic"[MeSH Terms] OR ("central"[All Fields] AND "african"[All Fields] AND "republic"[All Fields]) OR "central african republic"[All Fields]) OR ("yemen"[MeSH Terms] OR "yemen"[All Fields]) OR ("ethiopia"[MeSH Terms] OR "ethiopia"[All Fields] OR "ethiopia s"[All Fields]) OR ("niger"[MeSH Terms] OR "niger"[All Fields]) OR ("gambia"[MeSH Terms] OR "gambia"[All Fields] OR "gambia s"[All Fields]) OR ("rwanda"[MeSH Terms] OR "rwanda"[All Fields] OR "rwanda s"[All Fields]) OR ("guinea"[MeSH Terms] OR "guinea"[All Fields] OR "guinea s"[All Fields] OR "guineas"[All Fields]) OR ("sierra leone"[MeSH Terms] OR ("sierra"[All Fields] AND "leone"[All Fields]) OR "sierra leone"[All Fields]) OR ("angola"[MeSH Terms] OR "angola"[All Fields] OR "angola s"[All Fields]) OR ("honduras"[MeSH | | |

| | | | |
|---|---|---|---|
| | Terms] OR "honduras"[All Fields]) OR ("philippine"[All Fields] OR "philippines"[MeSH Terms] OR "philippines"[All Fields]) OR ("algeria"[MeSH Terms] OR "algeria"[All Fields]) OR ("india"[MeSH Terms] OR "india"[All Fields] OR "india s"[All Fields] OR "indias"[All Fields]) OR ("samoa"[MeSH Terms] OR "samoa"[All Fields] OR "samoas"[All Fields]) OR ("bangladesh"[MeSH Terms] OR "bangladesh"[All Fields] OR "bangladesh s"[All Fields]) OR ("indonesia"[MeSH Terms] OR "indonesia"[All Fields] OR "indonesia s"[All Fields] OR "indonesias"[All Fields]) OR ("sao"[All Fields] AND "tome"[All Fields]) OR ("principe"[All Fields] OR "principes"[All Fields]) OR ("belize"[MeSH Terms] OR "belize"[All Fields]) OR ("iran"[MeSH Terms] OR "iran"[All Fields]) OR ("senegal"[MeSH Terms] OR "senegal"[All Fields] OR "senegal s"[All Fields]) OR ("benin"[MeSH Terms] OR "benin"[All Fields] OR "benin s"[All Fields]) OR ("kenya"[MeSH Terms] OR "kenya"[All Fields] OR "kenya s"[All Fields]) OR ("melanesia"[MeSH Terms] OR "melanesia"[All Fields] OR ("solomon"[All Fields] AND "islands"[All Fields]) OR "solomon islands"[All Fields]) OR ("bhutan"[MeSH Terms] OR "bhutan"[All Fields] OR "bhutan s"[All Fields]) OR ("micronesia"[MeSH Terms] OR "micronesia"[All Fields] OR "kiribati"[All Fields]) OR ("sri lanka"[MeSH Terms] OR ("sri"[All Fields] AND "lanka"[All Fields]) OR "sri lanka"[All Fields]) OR ("bolivia"[MeSH Terms] OR "bolivia"[All Fields]) OR ("kyrgyzstan"[MeSH Terms] OR "kyrgyzstan"[All Fields] OR ("kyrgyz"[All Fields] AND "republic"[All Fields]) OR "kyrgyz republic"[All Fields]) OR ("lao"[All Fields] AND "pdr"[All Fields]) OR ("tajikistan"[MeSH Terms] OR "tajikistan"[All Fields]) OR ("cambodia"[MeSH Terms] OR "cambodia"[All Fields] OR "cambodia s"[All Fields]) OR ("lesotho"[MeSH Terms] OR "lesotho"[All Fields]) OR ("timor leste"[MeSH Terms] OR "timor leste"[All Fields] OR ("timor"[All Fields] AND "leste"[All Fields]) OR "timor leste"[All Fields]) OR ("cameroon"[MeSH Terms] OR "cameroon"[All Fields] OR "cameroons"[All Fields] OR "cameroon s"[All Fields]) OR ("mauritania"[MeSH Terms] OR "mauritania"[All Fields]) OR ("tunisia"[MeSH Terms] OR "tunisia"[All Fields]) OR ("comoros"[MeSH Terms] OR "comoros"[All Fields] OR "comoro"[All Fields]) OR ("micronesia"[MeSH Terms] OR "micronesia"[All Fields]) OR ("ukraine"[MeSH Terms] OR "ukraine"[All Fields] OR "ukraine s"[All Fields]) OR ("congo"[MeSH Terms] OR "congo"[All Fields]) OR ("mongolia"[MeSH Terms] OR "mongolia"[All Fields] OR "mongolia s"[All Fields]) OR ("uzbekistan"[MeSH Terms] OR "uzbekistan"[All Fields]) OR ("cote d ivoire"[MeSH Terms] OR ("cote"[All Fields] AND "d ivoire"[All Fields]) OR "cote d ivoire"[All Fields]) OR ("morocco"[MeSH Terms] OR "morocco"[All Fields]) OR ("vanuatu"[MeSH Terms] OR "vanuatu"[All Fields]) OR ("djibouti"[MeSH Terms] OR "djibouti"[All Fields]) OR ("myanmar"[MeSH Terms] OR "myanmar"[All Fields] OR "myanmar s"[All Fields] OR "myanmars"[All Fields]) OR ("vietnam"[MeSH Terms] OR "vietnam"[All Fields] OR "vietnam s"[All Fields]) OR ("egypt"[MeSH Terms] OR "egypt"[All Fields] OR "egypt s"[All Fields]) OR ("nepal"[MeSH Terms] OR "nepal"[All Fields] | | |

| | | | |
|---|---|---|---|
| | OR "nepal s"[All Fields]) OR ("middle east"[MeSH Terms] OR ("middle"[All Fields] AND "east"[All Fields]) OR "middle east"[All Fields] OR ("west"[All Fields] AND "bank"[All Fields]) OR "west bank"[All Fields]) OR "gaza"[All Fields] OR ("brazil"[MeSH Terms] OR "brazil"[All Fields] OR "brazil s"[All Fields] OR "brazils"[All Fields]) OR ("kazakhstan"[MeSH Terms] OR "kazakhstan"[All Fields] OR "kazakhstan s"[All Fields]) OR ("south africa"[MeSH Terms] OR ("south"[All Fields] AND "africa"[All Fields]) OR "south africa"[All Fields]) OR ("bulgaria"[MeSH Terms] OR "bulgaria"[All Fields]) OR ("kosovo"[MeSH Terms] OR "kosovo"[All Fields] OR "kosovo s"[All Fields]) OR ("saint lucia"[MeSH Terms] OR ("saint"[All Fields] AND "lucia"[All Fields]) OR "saint lucia"[All Fields] OR ("st"[All Fields] AND "lucia"[All Fields]) OR "st lucia"[All Fields]) OR ("china"[MeSH Terms] OR "china"[All Fields] OR "china s"[All Fields] OR "chinas"[All Fields]) OR ("lebanon"[MeSH Terms] OR "lebanon"[All Fields] OR "lebanon s"[All Fields]) OR (vincent, st[Investigator] OR st vincent[Author] OR st vincent[Investigator]) OR ("saint vincent and the grenadines"[MeSH Terms] OR ("saint"[All Fields] AND "vincent"[All Fields] AND "grenadines"[All Fields]) OR "saint vincent and the grenadines"[All Fields] OR "grenadines"[All Fields]) OR ("colombia"[MeSH Terms] OR "colombia"[All Fields] OR "colombia s"[All Fields]) OR ("libya"[MeSH Terms] OR "libya"[All Fields]) OR ("suriname"[MeSH Terms] OR "suriname"[All Fields] OR "surinam"[All Fields]) OR ("costa rica"[MeSH Terms] OR ("costa"[All Fields] AND "rica"[All Fields]) OR "costa rica"[All Fields]) OR ("malaysia"[MeSH Terms] OR "malaysia"[All Fields] OR "malaysia s"[All Fields]) OR ("dominican republic"[MeSH Terms] OR ("dominican"[All Fields] AND "republic"[All Fields]) OR "dominican republic"[All Fields]) OR ("mauritius"[MeSH Terms] OR "mauritius"[All Fields]) OR ("turkmenistan"[MeSH Terms] OR "turkmenistan"[All Fields]) OR ("equatorial guinea"[MeSH Terms] OR ("equatorial"[All Fields] AND "guinea"[All Fields]) OR "equatorial guinea"[All Fields]) OR ("mexico"[MeSH Terms] OR "mexico"[All Fields] OR "mexico s"[All Fields] OR "mexicos"[All Fields]) OR ("micronesia"[MeSH Terms] OR "micronesia"[All Fields] OR "tuvalu"[All Fields]) OR ("ecuador"[MeSH Terms] OR "ecuador"[All Fields] OR "ecuador s"[All Fields]) OR ("moldova"[MeSH Terms] OR "moldova"[All Fields]) OR ("fiji"[MeSH Terms] OR "fiji"[All Fields]) OR ("montenegro"[MeSH Terms] OR "montenegro"[All Fields]) OR ("afghanistan"[MeSH Terms] OR "afghanistan"[All Fields] OR "afghanistan s"[All Fields]) OR ("guinea bissau"[MeSH Terms] OR "guinea bissau"[All Fields] OR ("guinea"[All Fields] AND "bissau"[All Fields]) OR "guinea bissau"[All Fields]) OR ("somalia"[MeSH Terms] OR "somalia"[All Fields]) OR ("burkina faso"[MeSH Terms] OR ("burkina"[All Fields] AND "faso"[All Fields]) OR "burkina faso"[All Fields]) OR ("korea"[MeSH Terms] OR "korea"[All Fields] OR "korea s"[All Fields] OR "koreas"[All Fields]) OR ("south sudan"[MeSH Terms] OR ("south"[All Fields] AND "sudan"[All Fields]) OR "south sudan"[All Fields]) OR | | |

| | | | |
|---|---|---|---|
| | ("burundi"[MeSH Terms] OR "burundi"[All Fields]) OR ("liberia"[MeSH Terms] OR "liberia"[All Fields] OR "liberia s"[All Fields]) OR ("sudan"[MeSH Terms] OR "sudan"[All Fields] OR "sudans"[All Fields] OR "sudan s"[All Fields]) OR ("madagascar"[MeSH Terms] OR "madagascar"[All Fields] OR "madagascar s"[All Fields]) OR ("syria"[MeSH Terms] OR "syria"[All Fields] OR "syria s"[All Fields]) OR ("chad"[MeSH Terms] OR "chad"[All Fields]) OR ("malawi"[MeSH Terms] OR "malawi"[All Fields] OR "malawi s"[All Fields]) OR ("togo"[MeSH Terms] OR "togo"[All Fields]) OR ("congo"[MeSH Terms] OR "congo"[All Fields]) OR ("dem"[All Fields] AND "rep"[All Fields]) OR ("mali"[MeSH Terms] OR "mali"[All Fields]) OR ("uganda"[MeSH Terms] OR "uganda"[All Fields] OR "uganda s"[All Fields]) OR ("eritrea"[MeSH Terms] OR "eritrea"[All Fields]) OR ("mozambique"[MeSH Terms] OR "mozambique"[All Fields] OR "mozambique s"[All Fields]) OR ("ethiopia"[MeSH Terms] OR "ethiopia"[All Fields] OR "ethiopia s"[All Fields]) OR ("niger"[MeSH Terms] OR "niger"[All Fields]) OR ("gambia"[MeSH Terms] OR "gambia"[All Fields] OR "gambia s"[All Fields]) OR ("rwanda"[MeSH Terms] OR "rwanda"[All Fields] OR "rwanda s"[All Fields]) OR (("guinea"[MeSH Terms] OR "guinea"[All Fields] OR "guinea s"[All Fields] OR "guineas"[All Fields]) AND ("sierra leone"[MeSH Terms] OR ("sierra"[All Fields] AND "leone"[All Fields]) OR "sierra leone"[All Fields])) OR ("angola"[MeSH Terms] OR "angola"[All Fields] OR "angola s"[All Fields]) OR ("honduras"[MeSH Terms] OR "honduras"[All Fields]) OR ("philippine"[All Fields] OR "philippines"[MeSH Terms] OR "philippines"[All Fields]) OR ("algeria"[MeSH Terms] OR "algeria"[All Fields]) OR ("india"[MeSH Terms] OR "india"[All Fields] OR "india s"[All Fields] OR "indias"[All Fields]) OR ("samoa"[MeSH Terms] OR "samoa"[All Fields] OR "samoas"[All Fields]) OR ("bangladesh"[MeSH Terms] OR "bangladesh"[All Fields] OR "bangladesh s"[All Fields]) OR ("indonesia"[MeSH Terms] OR "indonesia"[All Fields] OR "indonesia s"[All Fields] OR "indonesias"[All Fields]) OR ("sao"[All Fields] AND "tome"[All Fields]) OR ("principe"[All Fields] OR "principes"[All Fields]) OR ("tanzania"[MeSH Terms] OR "tanzania"[All Fields] OR "tanzania s"[All Fields]) OR ("cabo verde"[MeSH Terms] OR ("cabo"[All Fields] AND "verde"[All Fields]) OR "cabo verde"[All Fields]) OR ("eswatini"[MeSH Terms] OR "eswatini"[All Fields]) OR ("nigeria"[MeSH Terms] OR "nigeria"[All Fields] OR "nigeria s"[All Fields]) OR ("zimbabwe"[MeSH Terms] OR "zimbabwe"[All Fields] OR "zimbabwe s"[All Fields]) OR ("ghana"[MeSH Terms] OR "ghana"[All Fields] OR "ghana s"[All Fields]) OR ("pakistan"[MeSH Terms] OR "pakistan"[All Fields] OR "pakistan s"[All Fields]) OR ("haiti"[MeSH Terms] OR "haiti"[All Fields] OR "haiti s"[All Fields]) OR ("papua new guinea"[MeSH Terms] OR ("papua"[All Fields] AND "new"[All Fields] AND "guinea"[All Fields]) OR "papua new guinea"[All Fields]) OR ("albania"[MeSH Terms] OR "albania"[All Fields]) OR ("namibia"[MeSH Terms] OR "namibia"[All Fields]) OR ("american samoa"[MeSH Terms] OR ("american"[All Fields] | | |

| | | | |
|---|---|---|---|
| | AND "samoa"[All Fields]) OR "american samoa"[All Fields]) OR ("georgia"[MeSH Terms] OR "georgia"[All Fields] OR "georgia republic"[MeSH Terms] OR ("georgia"[All Fields] AND "republic"[All Fields]) OR "georgia republic"[All Fields] OR "georgia s"[All Fields]) OR ("gabon"[MeSH Terms] OR "gabon"[All Fields]) OR ("republic of north macedonia"[MeSH Terms] OR ("republic"[All Fields] AND "north"[All Fields] AND "macedonia"[All Fields]) OR "republic of north macedonia"[All Fields] OR ("north"[All Fields] AND "macedonia"[All Fields]) OR "north macedonia"[All Fields]) OR ("argentina"[MeSH Terms] OR "argentina"[All Fields] OR "argentina s"[All Fields] OR "argentinae"[All Fields]) OR ("grenada"[MeSH Terms] OR "grenada"[All Fields]) OR ("panama"[MeSH Terms] OR "panama"[All Fields] OR "panama s"[All Fields]) OR ("armenia"[MeSH Terms] OR "armenia"[All Fields]) OR ("guatemala"[MeSH Terms] OR "guatemala"[All Fields] OR "guatemala s"[All Fields]) OR ("paraguai"[All Fields] OR "paraguay"[MeSH Terms] OR "paraguay"[All Fields]) OR ("el salvador"[MeSH Terms] OR ("el"[All Fields] AND "salvador"[All Fields]) OR "el salvador"[All Fields]) OR ("nicaragua"[MeSH Terms] OR "nicaragua"[All Fields] OR "nicaragua s"[All Fields]) OR ("zambia"[MeSH Terms] OR "zambia"[All Fields] OR "zambia s"[All Fields]) OR ("azerbaijan"[MeSH Terms] OR "azerbaijan"[All Fields]) OR ("guyana"[MeSH Terms] OR "guyana"[All Fields]) OR ("peru"[MeSH Terms] OR "peru"[All Fields]) OR ("republic of belarus"[MeSH Terms] OR ("republic"[All Fields] AND "belarus"[All Fields]) OR "republic of belarus"[All Fields] OR "belarus"[All Fields]) OR ("iraq"[MeSH Terms] OR "iraq"[All Fields]) OR ("romania"[MeSH Terms] OR "romania"[All Fields] OR "romania s"[All Fields]) OR ("bosnia and herzegovina"[MeSH Terms] OR ("bosnia"[All Fields] AND "herzegovina"[All Fields]) OR "bosnia and herzegovina"[All Fields] OR "bosnia"[All Fields]) OR ("bosnia and herzegovina"[MeSH Terms] OR ("bosnia"[All Fields] AND "herzegovina"[All Fields]) OR "bosnia and herzegovina"[All Fields] OR "herzegovina"[All Fields]) OR ("jamaica"[MeSH Terms] OR "jamaica"[All Fields] OR "jamaica s"[All Fields]) OR ("russia"[MeSH Terms] OR "russia"[All Fields] OR "russia s"[All Fields] OR "russias"[All Fields]) OR ("botswana"[MeSH Terms] OR "botswana"[All Fields] OR "botswana s"[All Fields]) OR ("jordan"[MeSH Terms] OR "jordan"[All Fields]) OR ("serbia"[MeSH Terms] OR "serbia"[All Fields]) OR ("thailand"[MeSH Terms] OR "thailand"[All Fields] OR "thailand s"[All Fields]) OR ("cuba"[MeSH Terms] OR "cuba"[All Fields]) OR ("indian ocean islands"[MeSH Terms] OR ("indian"[All Fields] AND "ocean"[All Fields] AND "islands"[All Fields]) OR "indian ocean islands"[All Fields] OR "maldives"[All Fields] OR "maldive"[All Fields]) OR ("tonga"[MeSH Terms] OR "tonga"[All Fields] OR "tonga s"[All Fields]) OR ("dominica"[MeSH Terms] OR "dominica"[All Fields]) OR ("micronesia"[MeSH Terms] OR "micronesia"[All Fields] OR ("marshall"[All Fields] AND "islands"[All Fields]) OR "marshall islands"[All Fields]) OR ("turkey"[MeSH Terms] OR "turkey"[All Fields] OR "turkey s"[All Fields] OR | | |

| | | | |
|---|---|---|---|
| | "turkeys"[MeSH Terms] OR "turkeys"[All Fields]) OR ("equatorial guinea"[MeSH Terms] OR ("equatorial"[All Fields] AND "guinea"[All Fields]) OR "equatorial guinea"[All Fields]) OR ("mexico"[MeSH Terms] OR "mexico"[All Fields] OR "mexico s"[All Fields] OR "mexicos"[All Fields]) OR (("micronesia"[MeSH Terms] OR "micronesia"[All Fields] OR "tuvalu"[All Fields]) AND ("ecuador"[MeSH Terms] OR "ecuador"[All Fields] OR "ecuador s"[All Fields])) OR "developing countries"[MeSH Terms] OR "developing countries"[MeSH Terms] OR "developing countries"[MeSH Terms] OR "developing countries"[MeSH Terms] OR "afghanistan"[MeSH Terms] OR "albania"[MeSH Terms] OR "algeria"[MeSH Terms] OR "argentina"[MeSH Terms] OR "armenia"[MeSH Terms] OR "angola"[MeSH Terms] OR "american samoa"[MeSH Terms] OR "azerbaijan"[MeSH Terms] OR "bangladesh"[MeSH Terms] OR "republic of belarus"[MeSH Terms] OR "belize"[MeSH Terms] OR "benin"[MeSH Terms] OR "bhutan"[MeSH Terms] OR "bolivia"[MeSH Terms] OR "bosnia and herzegovina"[MeSH Terms] OR "botswana"[MeSH Terms] OR "brazil"[MeSH Terms] OR "bulgaria"[MeSH Terms] OR "burkina faso"[MeSH Terms] OR "burundi"[MeSH Terms] OR "cabo verde"[MeSH Terms] OR "cambodia"[MeSH Terms] OR "cameroon"[MeSH Terms] OR "central african republic"[MeSH Terms] OR "chad"[MeSH Terms] OR "china"[MeSH Terms] OR "colombia"[MeSH Terms] OR "comoros"[MeSH Terms] OR "congo"[MeSH Terms] OR "congo"[MeSH Terms] OR "costa rica"[MeSH Terms] OR "cote d ivoire"[MeSH Terms] OR "cuba"[MeSH Terms] OR "djibouti"[MeSH Terms] OR "dominica"[MeSH Terms] OR "dominican republic"[MeSH Terms] OR "ecuador"[MeSH Terms] OR "egypt"[MeSH Terms] OR "el salvador"[MeSH Terms] OR "equatorial guinea"[MeSH Terms] OR "eritrea"[MeSH Terms] OR "eswatini"[MeSH Terms] OR "ethiopia"[MeSH Terms] OR "fiji"[MeSH Terms] OR "gabon"[MeSH Terms] OR "gambia"[MeSH Terms] OR ("georgia"[MeSH Terms] OR "georgia republic"[MeSH Terms]) OR "ghana"[MeSH Terms] OR "grenada"[MeSH Terms] OR "guatemala"[MeSH Terms] OR "guinea"[MeSH Terms] OR "guinea bissau"[MeSH Terms] OR "guyana"[MeSH Terms] OR "haiti"[MeSH Terms] OR "bosnia and herzegovina"[MeSH Terms] OR "honduras"[MeSH Terms] OR "india"[MeSH Terms] OR "indonesia"[MeSH Terms] OR "iran"[MeSH Terms] OR "iraq"[MeSH Terms] OR "jamaica"[MeSH Terms] OR "jordan"[MeSH Terms] OR "kazakhstan"[MeSH Terms] OR "kenya"[MeSH Terms] OR "micronesia"[MeSH Terms] OR "korea"[MeSH Terms] OR "kosovo"[MeSH Terms] OR "kyrgyzstan"[MeSH Terms] OR "lebanon"[MeSH Terms] OR "lesotho"[MeSH Terms] OR "liberia"[MeSH Terms] OR "libya"[MeSH Terms] OR "madagascar"[MeSH Terms] OR "malawi"[MeSH Terms] OR "malaysia"[MeSH Terms] OR "indian ocean islands"[MeSH Terms] OR "mali"[MeSH Terms] OR "micronesia"[MeSH Terms] OR "mauritania"[MeSH Terms] OR "mauritius"[MeSH Terms] OR "mexico"[MeSH Terms] OR "micronesia"[MeSH Terms] OR "moldova"[MeSH Terms] OR "mongolia"[MeSH Terms] OR "montenegro"[MeSH | | |

| | | | |
|---|---|---|---|
| | Terms] OR "morocco"[MeSH Terms] OR "mozambique"[MeSH Terms] OR "myanmar"[MeSH Terms] OR "namibia"[MeSH Terms] OR "nepal"[MeSH Terms] OR "nicaragua"[MeSH Terms] OR "niger"[MeSH Terms] OR "nigeria"[MeSH Terms] OR "republic of north macedonia"[MeSH Terms] OR "pakistan"[MeSH Terms] OR "panama"[MeSH Terms] OR "papua new guinea"[MeSH Terms] OR "paraguay"[MeSH Terms] OR "peru"[MeSH Terms] OR "philippines"[MeSH Terms] OR "romania"[MeSH Terms] OR "russia"[MeSH Terms] OR "rwanda"[MeSH Terms] OR "samoa"[MeSH Terms] OR "senegal"[MeSH Terms] OR "serbia"[MeSH Terms] OR "sierra leone"[MeSH Terms] OR "melanesia"[MeSH Terms] OR "somalia"[MeSH Terms] OR "south africa"[MeSH Terms] OR "south sudan"[MeSH Terms] OR "saint lucia"[MeSH Terms] OR "sudan"[MeSH Terms] OR "suriname"[MeSH Terms] OR "sri lanka"[MeSH Terms] OR "syria"[MeSH Terms] OR "tajikistan"[MeSH Terms] OR "tanzania"[MeSH Terms] OR "thailand"[MeSH Terms] OR ("saint vincent and the grenadines"[MeSH Terms]) OR "timor leste"[MeSH Terms] OR "togo"[MeSH Terms] OR "tonga"[MeSH Terms] OR "tunisia"[MeSH Terms] OR "turkey"[MeSH Terms] OR "turkmenistan"[MeSH Terms] OR "micronesia"[MeSH Terms] OR "uganda"[MeSH Terms] OR "ukraine"[MeSH Terms] OR "uzbekistan"[MeSH Terms] OR "vanuatu"[MeSH Terms] OR "vietnam"[MeSH Terms] OR "middle east"[MeSH Terms] OR "yemen"[MeSH Terms] OR "zambia"[MeSH Terms] OR "zimbabwe"[MeSH Terms]) AND (("under five"[All Fields] AND ("child"[MeSH Terms] OR "child"[All Fields] OR "children"[All Fields] OR "child s"[All Fields] OR "children s"[All Fields] OR "childrens"[All Fields] OR "childs"[All Fields])) OR ("child"[MeSH Terms] OR "child"[All Fields] OR "children"[All Fields] OR "child s"[All Fields] OR "children s"[All Fields] OR "childrens"[All Fields] OR "childs"[All Fields]) OR ("infant"[MeSH Terms] OR "infant"[All Fields] OR "infants"[All Fields] OR "infant s"[All Fields]) OR ("child, preschool"[MeSH Terms] OR ("child"[All Fields] AND "preschool"[All Fields]) OR "preschool child"[All Fields] OR "preschooler"[All Fields] OR "preschoolers"[All Fields] OR "preschool"[All Fields] OR "preschooler s"[All Fields] OR "preschools"[All Fields]) OR ("toddler"[All Fields] OR "toddler s"[All Fields] OR "toddlers"[All Fields]) OR ("childhood"[All Fields] OR "childhoods"[All Fields]) OR "child"[MeSH Terms] OR "infant"[MeSH Terms] OR "child"[MeSH Terms]) | | |

Logic Grid and search terms for Web of Science for systematic review of incidence of diarrhea among under-five children in low- and Middle-income countries.

| **Condition** | **Context** | | **Population** |
|---|---|---|---|
| **Text words** | **Text words** | | **Text words** |
| • Diarrhea<br>• Dysentery<br>• Acute diarrheal disease<br>• Prevalence<br>• Incidence<br>• Incidence study<br>• Incidence rate<br>• Cumulative incidence<br>• Frequency<br>• Monitoring<br>• disease burden<br>• Acute diarrhea<br>• Acute Diarrheal Disease in Children | • Low-income countries<br>• Middle income countries<br>• Developing country<br>• Least Developed Countries<br>• Less Developed Countries<br>• Third-World Countries<br>• Low-Income Countries<br>• Central African Republic<br>• Afghanistan<br>• Albania<br>• Algeria<br>• Algeria<br>• American Samoa<br>• Angola<br>• Angola<br>• Argentina<br>• Armenia<br>• Azerbaijan<br>• Bangladesh<br>• Bangladesh<br>• Belarus<br>• Belize<br>• Benin<br>• Bhutan<br>• Bolivia<br>• Bosnia<br>• Botswana<br>• Brazil<br>• Bulgaria<br>• Burkina Faso<br>• Burundi<br>• Cabo Verde<br>• Cambodia<br>• Cameroon<br>• Chad<br>• China | • Congo<br>• Congo<br>• Costa Rica<br>• Côte d'Ivoire<br>• Cuba<br>• Dem. Rep<br>• Djibouti<br>• Dominica<br>• Dominican Republic<br>• Ecuador<br>• Ecuador<br>• Egypt<br>• El Salvador<br>• Equatorial Guinea<br>• Equatorial Guinea<br>• Eritrea<br>• Eswatini<br>• Ethiopia<br>• Ethiopia<br>• Fiji<br>• Gabon<br>• Gambia<br>• Gambia<br>• Gaza<br>• Georgia<br>• Ghana<br>• Grenada<br>• Guatemala<br>• Guinea<br>• Guinea<br>• Guinea-Bissau<br>• Guyana<br>• Haiti<br>• Herzegovina<br>• Honduras<br>• Honduras | • Under-five children<br>• Child<br>• Infant<br>• Preschool<br>• Toddler<br>• Childhood |

| | | | |
|---|---|---|---|
| | • Colombia<br>• Comoros<br>• Liberia<br>• Libya<br>• Madagascar<br>• Malawi<br>• Malaysia<br>• Maldives<br>• Mali<br>• Marshall Islands<br>• Mauritania<br>• Mauritius<br>• Mexico<br>• Mexico<br>• Micronesia<br>• Moldova<br>• Mongolia<br>• Montenegro<br>• Morocco<br>• Mozambique<br>• Myanmar<br>• Namibia<br>• Nepal<br>• Nicaragua<br>• Niger<br>• Niger<br>• Nigeria<br>• North Macedonia<br>• Pakistan<br>• Panama<br>• Papua New Guinea<br>• Paraguay<br>• Peru<br>• Philippines<br>• Philippines<br>• Principe<br>• Principe<br>• Romania<br>• Russia<br>• Rwanda<br>• Rwanda<br>• Samoa<br>• Samoa<br>• São Tomé | • India<br>• India<br>• Indonesia<br>• Indonesia<br>• Iran<br>• Iraq<br>• Jamaica<br>• Jordan<br>• Kazakhstan<br>• Kenya<br>• Kiribati<br>• Korea<br>• Kosovo<br>• Kyrgyz Republic<br>• Lao PDR<br>• Lebanon<br>• Lesotho<br>• Serbia<br>• Sierra Leone<br>• Sierra Leone<br>• Solomon Islands<br>• Somalia<br>• South Africa<br>• South Sudan<br>• Sri Lanka<br>• St. Lucia<br>• St. Vincent<br>• Sudan<br>• Suriname<br>• Syria<br>• Tajikistan<br>• Tanzania<br>• Thailand<br>• The Grenadines<br>• Timor-Leste<br>• Togo<br>• Tonga<br>• Tunisia<br>• Turkey<br>• Turkmenistan<br>• Tuvalu<br>• Tuvalu<br>• Uganda<br>• Ukraine | |

| | | | |
|---|---|---|---|
| | • São Tomé<br>• Senegal<br>• Yemen<br>• Zambia<br>• Zimbabwe | • Uzbekistan<br>• Vanuatu<br>• Vietnam<br>• West Bank | |

**Search history results from Web of Science** for systematic review of incidence of diarrhea among under-five children in low- and Middle-income countries.

| Search # | Search terms (filter applied From 2010/9/1 to 2022/2/21) | Search results | Date & time |
|---|---|---|---|
| #1 | Condition<br><br>((((((((((((ALL=(Diarrhea)) OR ALL=(Dysentery )) OR ALL=(Acute diarrheal disease )) OR ALL=(Prevalence)) OR ALL=(Incidence )) OR ALL=(Incidence study )) OR ALL=(Incidence rate )) OR ALL=(Cumulative incidence )) OR ALL=(Frequency )) OR ALL=(Monitoring )) OR ALL=(disease burden )) OR ALL=(Acute diarrhea )) OR ALL=(Acute Diarrheal Disease in Children ) | **3,520,730** | February 23, 2022 |
| #2 | Context<br><br>((((((((((((((((((((((((((((((((((((((((((((((((ALL=(Low-income countries )) OR ALL=(Middle income countries)) OR ALL=(Developing country )) OR ALL=(Least Developed Countries )) OR ALL=(Less Developed Countries )) OR ALL=(Third-World Countries)) OR ALL=(Low-Income Countries )) OR ALL=(Central African Republic)) OR ALL=(Afghanistan )) OR ALL=(Albania )) OR ALL=(Algeria)) OR ALL=(American Samoa )) OR ALL=(Angola )) OR ALL=(Argentina )) OR ALL=(Armenia )) OR ALL=(Azerbaijan )) OR ALL=(Bangladesh)) OR ALL=(Belarus )) OR ALL=(Belize )) OR ALL=(Benin)) OR ALL=(Bhutan )) OR ALL=(Bolivia )) OR ALL=(Bosnia)) OR ALL=(Botswana )) OR ALL=(Brazil )) OR ALL=(Bulgaria)) OR ALL=(Burkina Faso)) OR ALL=( Burundi)) OR ALL=(Cabo Verde )) OR ALL=(Cambodia )) OR ALL=(Cameroon)) OR ALL=(Chad )) OR ALL=(China )) OR ALL=(Colombia )) OR ALL=(Comoros)) OR ALL=(Congo )) OR ALL=(Costa Rica )) OR ALL=(Côte d'Ivoire)) OR ALL=(Cuba )) OR ALL=(Congo Republic )) OR ALL=(Djibouti )) OR ALL=(Dominica)) OR ALL=(Dominican Republic )) OR ALL=(Ecuador )) OR ALL=(Egypt)) OR ALL=(El Salvador )) OR ALL=(Equatorial Guinea)) OR ALL=(Eritrea)) OR ALL=(Eswatini)) OR ALL=(Ethiopia ) | **7,517,571** | February 23, 2022 |
| #3 | Context | | |

| | | | |
|---|---|---|---|
| | (((((((((((((((((((((((((((((((((((((((((((((((((((((((((((((ALL=(Fiji )) OR ALL=(Gabon )) OR ALL=(Gambia)) OR ALL=(Gaza )) OR ALL=(Georgia )) OR ALL=(Ghana)) OR ALL=(Grenada )) OR ALL=(Guatemala )) OR ALL=(Guinea)) OR ALL=(Guinea-Bissau )) OR ALL=(Guyana )) OR ALL=(Haiti)) OR ALL=(Herzegovina )) OR ALL=(Honduras )) OR ALL=(India)) OR ALL=(Indonesia )) OR ALL=(Iran)) OR ALL=(Iraq)) OR ALL=(Jamaica )) OR ALL=(Jordan )) OR ALL=(Kazakhstan )) OR ALL=(Kenya)) OR ALL=(Kiribati )) OR ALL=(Korea)) OR ALL=(Kosovo)) OR ALL=(Kyrgyz Republic )) OR ALL=(Lao PDR)) OR ALL=( Lebanon)) OR ALL=(Lesotho )) OR ALL=(Liberia )) OR ALL=(Libya)) OR ALL=(Madagascar )) OR ALL=(Malawi )) OR ALL=(Malaysia)) OR ALL=(Maldives )) OR ALL=(Mali )) OR ALL=(Marshall Islands)) OR ALL=(Mauritania )) OR ALL=(Mauritius )) OR ALL=(Mexico)) OR ALL=(Micronesia )) OR ALL=(Moldova )) OR ALL=(Mongolia )) OR ALL=(Montenegro )) OR ALL=(Morocco )) OR ALL=(Mozambique )) OR ALL=(Myanmar)) OR ALL=(Namibia )) OR ALL=(Nepal)) OR ALL=(Nicaragua )) OR ALL=(Niger )) OR ALL=(Nigeria )) OR ALL=(North Macedonia)) OR ALL=(Pakistan )) OR ALL=(Panama )) OR ALL=(Papua New Guinea)) OR ALL=(Paraguay )) OR ALL=(Peru )) OR ALL=(Philippines)) OR ALL=(Principe )) OR ALL=(Romania )) OR ALL=(Russia) | **5,766,609** | |
| #4 | Countries<br><br>(((((((((((((((((((((((((((((((((((ALL=(Rwanda )) OR ALL=(Samoa )) OR ALL=(São Tomé)) OR ALL=(Senegal )) OR ALL=(Serbia )) OR ALL=(Sierra Leone)) OR ALL=(Solomon Islands )) OR ALL=(Somalia )) OR ALL=(South Africa)) OR ALL=(South Sudan )) OR ALL=(Sri Lanka )) OR ALL=(Saint Lucia)) OR ALL=(Saint Vincent )) OR ALL=(Sudan )) OR ALL=(Suriname )) OR ALL=(Syria)) OR ALL=(Tajikistan )) OR ALL=(Tanzania )) OR ALL=(Thailand)) OR ALL=(The Grenadines )) OR ALL=(Timor-Leste )) OR ALL=(Togo)) OR ALL=(Tonga )) OR ALL=(Tunisia )) OR ALL=(Turkey)) OR ALL=(Turkmenistan )) OR ALL=(Tuvalu )) OR ALL=(Uganda)) OR ALL=(Ukraine )) OR ALL=(Uzbekistan )) OR ALL=(Vanuatu)) OR ALL=(Vietnam )) OR ALL=(West Bank )) OR ALL=(Yemen )) OR ALL=(Zambia)) OR ALL=(Zimbabwe ) | **1,720,666** | |
